# Safety and Reproducibility of a Clinical Trial System Using Induced Blood Stage *Plasmodium vivax* Infection and Its Potential as a Model to Evaluate Malaria Transmission

**DOI:** 10.1371/journal.pntd.0005139

**Published:** 2016-12-08

**Authors:** Paul Griffin, Cielo Pasay, Suzanne Elliott, Silvana Sekuloski, Maggy Sikulu, Leon Hugo, David Khoury, Deborah Cromer, Miles Davenport, Jetsumon Sattabongkot, Karen Ivinson, Christian Ockenhouse, James McCarthy

**Affiliations:** 1 Clinical Tropical Medicine Laboratory, QIMR Berghofer, Brisbane, Australia; 2 Q-Pharm Pty Ltd, Brisbane, Australia; 3 Department of Medicine and Infectious Diseases, Mater Hospital and Mater Medical Research Institute, Brisbane, Australia; 4 The University of Queensland, Brisbane, Australia; 5 University of New South Wales, Sydney, Australia; 6 Mahidol Vivax Research Unit, Faculty of Tropical Medicine, Mahidol University, Bangkok, Thailand; 7 PATH, Malaria Vaccine Initiative, Washington, DC, United States; Johns Hopkins Bloomberg School of Public Health, UNITED STATES

## Abstract

**Background:**

Interventions to interrupt transmission of malaria from humans to mosquitoes represent an appealing approach to assist malaria elimination. A limitation has been the lack of systems to test the efficacy of such interventions before proceeding to efficacy trials in the field. We have previously demonstrated the feasibility of induced blood stage malaria (IBSM) infection with *Plasmodium vivax*. In this study, we report further validation of the IBSM model, and its evaluation for assessment of transmission of *P*. *vivax* to *Anopheles stephensi* mosquitoes.

**Methods:**

Six healthy subjects (three cohorts, n = 2 per cohort) were infected with *P*. *vivax* by inoculation with parasitized erythrocytes. Parasite growth was monitored by quantitative PCR, and gametocytemia by quantitative reverse transcriptase PCR (qRT-PCR) for the mRNA *pvs25*. Parasite multiplication rate (PMR) and size of inoculum were calculated by linear regression. Mosquito transmission studies were undertaken by direct and membrane feeding assays over 3 days prior to commencement of antimalarial treatment, and midguts of blood fed mosquitoes dissected and checked for presence of oocysts after 7–9 days.

**Results:**

The clinical course and parasitemia were consistent across cohorts, with all subjects developing mild to moderate symptoms of malaria. No serious adverse events were reported. Asymptomatic elevated liver function tests were detected in four of six subjects; these resolved without treatment. Direct feeding of mosquitoes was well tolerated. The estimated PMR was 9.9 fold per cycle. Low prevalence of mosquito infection was observed (1.8%; n = 32/1801) from both direct (4.5%; n = 20/411) and membrane (0.9%; n = 12/1360) feeds.

**Conclusion:**

The *P*. *vivax* IBSM model proved safe and reliable. The clinical course and PMR were reproducible when compared with the previous study using this model. The IBSM model presented in this report shows promise as a system to test transmission-blocking interventions. Further work is required to validate transmission and increase its prevalence.

**Trial Registration:**

Anzctr.org.au ACTRN12613001008718

## Author Summary

Blocking the transmission of malaria from infected individuals to mosquitoes is an appealing approach to malaria elimination. However, at present there is no reliable experimental model to test the efficacy of transmission blocking interventions. In this study, we assessed the safety and reproducibility of our clinical trial model, in which we inject blood cells infected with malaria parasites into healthy volunteers. Furthermore, we tested if our clinical trial model could be used as a tool to evaluate malaria transmission. We infected healthy volunteers with *Plasmodium vivax* parasites and monitored parasite growth by molecular methods. When we detected the parasite stage that is infective to mosquitoes (the sexual stage), blood from infected volunteers was fed to mosquitoes. Then, we investigated the presence of parasites in the midgut of mosquitoes. The results from this study show that our clinical trial model is safe and reproducible. Moreover, we observed low levels of transmission of the malaria parasite from infected volunteers to mosquitoes. We need to validate this finding and to optimize it to increase the rate of malaria transmission. Altogether, our clinical trial model seems to be a reliable system to assess interventions to block malaria transmission, which has enormous public health significance.

## Introduction

A renewed focus on malaria elimination has increased the priority of research towards development of interventions to block malaria transmission, including transmission blocking vaccines (TBVs). By interrupting transmission of malaria parasites to mosquito vectors, a reduction in the number of secondary infections in the community is expected. It is hoped that TBVs can play a significant role in total interruption of malaria transmission in endemic areas. Similarly, a number of drugs in development appear to have gametocytocidal and/or sporontocidal activity [1–3]. Deployment of transmission-blocking interventions is predicted to be highly efficacious in integrated programs aimed to achieve the goal of malaria elimination.

The further development of transmission-blocking interventions requires a reliable way to select the best candidates for clinical progression. While clinical trials in malaria-endemic areas represent the gold standard for establishing the efficacy of any intervention, undertaking such trials entails major logistic challenges. This is particularly the case for TBVs, where clinical and transmission endpoints, such as changes in the number of infected mosquitoes, would be difficult to measure reliably in field studies. Therefore, other means of defining the transmission blocking activity of antimalarial interventions are required.

Significant advances have been made to establish a safe and reproducible controlled human malaria infection (CHMI) system with *P*. *falciparum* for the study of candidate antimalarial drugs and vaccines. These systems include infection by sporozoites—either introduced by mosquito bites [4–8] or by injection of cryopreserved sporozoites [9–11]-, and induced blood stage malaria (IBSM) by intravenous inoculation of parasite asexual stages [12–14]. At QIMR Berghofer we have successfully conducted IBSM studies with *P*. *falciparum* to investigate the efficacy of new antimalarial drugs by monitoring parasite growth and clearance after challenge [13].

CHMI methodologies for *P*. *vivax* have developed relatively slowly for a number of reasons, including the inability to culture this species *in vitro*. Previous *P*. *vivax* challenges have utilized sporozoites produced by experimental infection of mosquitoes with blood from infected patients [15–17]. The *P*. *vivax* mosquito challenge system has the drawback of the risk of relapse due to the formation of hypnozoites that may not be easily eradicated despite primaquine therapy in subjects with certain CYP2D6 phenotypes [17,18].

Recently, we established a human malaria parasite blood stage *P*. *vivax* (HMPBS-*Pv*) bank by collecting blood from a donor naturally infected with *P*. *vivax*, and reported the *in vivo* safety and infectivity of this isolate in two human subjects [19]. In addition, the parasite gene transcript *pvs25*, which is present in mature stage V gametocytes, the life cycle stage infectious to mosquitoes, was detected [19] at a time predictable from understanding of the *P*. *vivax* life cycle [20]. This suggests the presence of gametocytemia, which implies that these subjects would have been infectious to mosquito vectors. This *P*. *vivax* IBSM model offers the potential to test efficacy of *P*. *vivax* vaccines and drugs in non-immune subjects in a rapid and cost effective manner, and therefore has the potential to accelerate the clinical development of treatments for *P*. *vivax* malaria. Furthermore, this system eliminates the risk for recurrent infections in healthy volunteers by excluding of the liver stage of the parasite, as well as facilitates infection, which is achieved by intravenous injection rather than mosquito feeding.

The primary aims of the present study were to evaluate the safety and reproducibility of the *P*. *vivax* IBSM model and to investigate infectivity to vector mosquitoes through the addition of transmission endpoints.

## Methods

### Study design

This was a phase I, single-center study in adult healthy volunteers. The study was conducted at the contract research organization Q-Pharm Pty Ltd (Queensland, Australia). Mosquito transmission experiments were conducted at the QIMR Berghofer PC3 Insectary.

### Ethics

This study was approved by the QIMR Berghofer Human Research Ethics Committee and the Western Institutional Review Board (WIRB). WIRB was the ethics board for PATH (the funding partner) for this study. The trial was registered on the Australian and New Zealand Clinical Trials Registry (ANZCTR), ACTRN12613001008718. All subjects gave written informed consent before being included in the study.

### Subjects

Healthy male or female subjects between 18 and 50 years of age who met the inclusion and exclusion criteria were considered eligible for the study. Subjects were required to be blood group A and Duffy antigen positive in accordance with the blood type of the parasite donor, and the requirement for Duffy antigen to permit *P*. *vivax* infection. Details of the inclusion and exclusion criteria are presented in the Supplementary Information (S2 Supporting Information).

### Inoculum preparation

The human malaria parasite blood stage *P*. *vivax* (HMPBS-*Pv*) used in this trial was derived from blood donated from a malaria patient [19]. To prepare the inoculum for each cohort, two 1 mL vials of the *P*. *vivax* bank were combined into a single cell suspension; this was diluted to the appropriate volume and dispensed aseptically into 2 mL syringes for individual subject administration. The remaining cell suspension was used to quantify the inoculum size by quantitative PCR (qPCR) [19]. Previous experience [19] had indicated that each subject was inoculated with 100 viable parasite-infected erythrocytes.

### Study conduct

After intravenous injection of parasites on study Day 0, subjects were monitored by daily telephone call for the first 6 days (see Schedule of Events in S1 Table). From Day 7, study subjects attended the clinical trial unit daily for blood collection to assess parasitemia by qPCR. Once PCR positive, twice daily monitoring of parasitemia by qPCR and gametocytemia by *pvs25* qRT-PCR was performed as well as assessment of unexpected early onset of symptoms or signs suggestive of malaria, or any adverse events (AEs). Mosquito transmission studies were undertaken on the 3 days prior to the anticipated commencement of treatment, which previous experience indicated would occur on Day 14 [19]. On each of the mosquito feeding days, blood samples were collected to monitor parasitemia by qPCR. As determined by clinically apparent evidence of malaria infection, subjects were admitted to the study unit and confined for safety monitoring and commencement of antimalarial treatment. Treatment consisted of six doses (12 hours apart) of artemether/lumefantrine (A/L; Riamet^®^, Novartis Pharmaceuticals Australia Pty Limited, Australia). Following A/L treatment, subjects were confined as inpatients for 36 hours to ensure tolerance of therapy and clinical response. If deemed clinically well, subjects were followed on an outpatient basis for continued dosing of A/L, as well as monitoring of safety and clearance of parasitemia. The end of study visit took place on Day 28, in which final clinical and safety assessments were undertaken.

### Safety assessment

Safety parameters measured in this study included physical examination, vital signs, ECGs and clinical laboratory tests. AEs were monitored via telephone, within the clinical research unit, and on an outpatient basis after malaria challenge inoculation and antimalarial drug administration. If an AE changed in severity, it was recorded as a new event to enable thorough tracking of event severity. Standard definitions of AE severity as used in clinical trials were applied [21]. Blood samples for laboratory tests and malaria monitoring were taken at screening and immediately prior to inoculation, and at nominated times after malaria challenge (S1 Table). Careful monitoring of the safety and tolerability of the mosquito feeding component was also undertaken.

### Parasitological endpoints

Growth and clearance of malaria parasites were evaluated by qPCR by targeting a 199 bp fragment of the *P*. *vivax* 18S rDNA gene utilizing a Taqman hydrolysis probe chemistry as previously described [19] with the times of sampling outlined in the schedule of events (S1 Table). Briefly, subjects were evaluated for the presence of a patent parasitemia by qPCR once per day from Day 7, then once first detected, twice daily until treatment. The parasitemia was followed until clearance was confirmed, and then repeated at the end of study visit. All samples were tested in duplicate during the study and retested after completion of the study. Gametocytemia was measured by *pvs25* qRT-PCR [19], beginning when qPCR results became positive.

### Estimates of the inoculum size, parasite growth rate and parasite multiplication rate (PMR)

The parasite growth rate and starting parasite concentration were estimated by regression using a linear-mixed effects model. The model was applied to the log-transformed parasite concentrations measured by qPCR prior to treatment. The limit of detection (LOD) was 10 parasites/mL. Negative PCR measurements and measurements of parasitemia below the LOD were set to 10 parasites/mL. However, we excluded negative measurements and measurements below the LOD before the first positive detection of parasites. The slope of the fitted linear regression model corresponds to the parasite growth rate, and the extrapolated y-intercept corresponds to the log of the starting inoculum size received by each individual. Subjects from the same cohort were assumed to have received the same starting concentration of parasites, since the inoculum administered to subjects in the same cohort was prepared from the same cell suspension. Therefore, we assumed that differences in starting parasite concentration for subjects in the same cohort were negligible. We then used model selection to assess whether there was any apparent variation in the inocula for each cohort, perhaps due to slight variations in the preparation process. A random effect in the growth rate for each subject was included in the model to capture differences between individuals. The analysis was performed using the ‘lme’ function from the ‘nlme’ (Linear and Nonlinear Mixed Effects Models) package in the statistical software package R (version 2.15.0). To determine whether there was a significant cohort effect in the growth rate or inoculum size, cohort was both included and excluded as a covariate in the linear model, and the best model was determined by comparing nested models using the chi-squared test and backwards elimination, and by comparing the log-likelihood of fitted non-nested models with the same number of parameters. The growth rate estimate from the regression model was transformed to estimate the PMR, using *PMR* = *e*^*L*×*g*^, where *L* is the cycle time of the parasite (days) and *g* is the estimated growth rate (day^-1^). The y-intercept estimate was transformed to estimate the starting concentration of parasites (*P*_0_), using *P*_0_ = *e*^*b*^, where *b* is the y-intercept (log parasites/mL). Due to the transformations applied, the errors in the estimates of PMR and growth rate are log-normally distributed. Hence, the confidence intervals (CI) of PMR and *P*_0_ were derived by applying the above transformations to the sampling distributions for growth rate and y-intercept.

### Transmission endpoints

#### Assessment of transmission of *P*. *vivax* from IBSM infected subjects to mosquitoes

Laboratory reared, 5–7 days old female *Anopheles stephensi* mosquitoes maintained in a controlled environment at the QIMR Berghofer insectary (27°C; relative humidity of 70% and 12:12 hour day: night light cycling with 30-minute dawn/dusk periods) were used in this study. Larvae were maintained at a density of approximately 300 larvae in 3 L of distilled water in plastic trays (42 × 36 × 6 cm) and fed ground TetraMin Tropical Fish Food Flakes (Tetra, Melle, Germany). Pupae were transferred to mosquito cages (30 × 30 × 30 cm) for adult emergence. Mosquitoes were starved overnight prior to conducting feeding assays. Mosquito feeding both by direct feeding assay (DFA, daily) and membrane feeding assay (MFA, twice daily) was conducted for 3 successive days prior to the anticipated treatment with antimalarials. For DFA, 30 *A*. *stephensi* female mosquitoes were allocated into 200 mL plastic feeding containers with gauze lids. Subjects were escorted to the insectary where a feeding container was placed on alternating sides of the volar surface of their forearms or thighs and mosquitoes allowed to feed for 10 ±5 minutes to enable them to fully engorge. For MFA, 5 mL of blood was collected from each subject into a heparinized vacutainer tube kept at 38°C for up to 10 minutes until dispensed into membrane feeders (to prevent premature exflagellation of *P*. *vivax* gametocytes prior to mosquito ingestion). Fifty *A*. *stephensi* female mosquitoes were placed in 750 mL plastic feeding containers with gauze lids. Mosquitoes were allowed to feed on the blood for 30 min via a membrane-feeding apparatus, in which blood was placed into the inner chamber of water jacketed glass feeders, covered on one end by bovine caecum membrane and maintained at 37°C by a recirculating water bath [22]. Mosquitoes fed by DFA on volunteers who were not infected with malaria were used as negative controls. Blood-fed mosquitoes were kept in an environmental chamber set at 26°C and relative humidity of 70% [23] and maintained with a 10% sugar solution supplemented with 0.05% para-amino benzoic acid to promote the sporogonic cycle [24]. Mosquitoes were dissected 7 to 9 days after blood feeding to examine for oocysts in midgut preparations. Midguts were stained with 0.5% mercurochrome in PBS for 5 min prior to visualization using a compound microscope.

#### Molecular and histological analyses of mosquitoes

Molecular and histological analyses of whole mosquitoes sampled from the mosquito colony cage (unfed) and negative control mosquitoes (fed on non-infected blood) were performed to identify atypical structures observed within mosquito midgut tissue. For molecular analysis of colony mosquitoes (n = 4), DNA was extracted from whole female *A*. *stephensi* according to a modification of the Livak protocol [25]. Midguts from negative control mosquitoes that showed atypical structures (n = 4) were recovered from the slides, gently homogenized in 50 μL QuickExtract DNA extraction solution (EPICENTRE Biotechnologies, Madison, WI, USA) and incubated according to the QuickExtract procedure. PCR detection for microsporidia known to infect *A*. *stephensi* was performed on the DNA extracts using three primer sets (*Nosema* group, *Pleistophora* group and *Brachiola algerae*) [26]. PCR products were extracted from gels, TA cloned in *E*. *coli* (dH5α strain) using the pGEM-T vector (Promega) according to the manufacturer’s instructions and DNA fragments were purified and sequenced by BigDye sequencing according to standard procedures. For histological analysis, 20 female *A*. *stephensi* from the colony cage were fixed in 4% paraformaldehyde, 0.5% Triton X and embedded in paraffin according to standard procedures. Paraffin sections (3–4 μM) were affixed to slides and air dried at 37°C. Periodic acid Schiff (PAS) and Gram chromotrope staining [27] were performed to test for the presence of fungal infection.

### Sample size

Based on previous published work undertaken during the malaria therapy for syphilis era [28], it was estimated that to demonstrate infectivity to vector mosquitoes, with a 95% probability of having at least one infected mosquito following DFA from the infected subject, the total number of human subjects required was six (with 90 mosquitoes feeding on every host; therefore, 30 mosquitoes/subject/day for up to 3 days). This calculation assumes a negative binomial distribution and that 40% or more of blood stage infections are transmissible to mosquitoes. The calculation is based on the mean infectivity data published for anopheline hosts infected with *P*. *vivax* [29,30]. Therefore, the study was designed to comprise six subjects, and conducted in three cohorts (n = 2 subjects per cohort).

## Results

### Subjects profile

**Fig 1:**
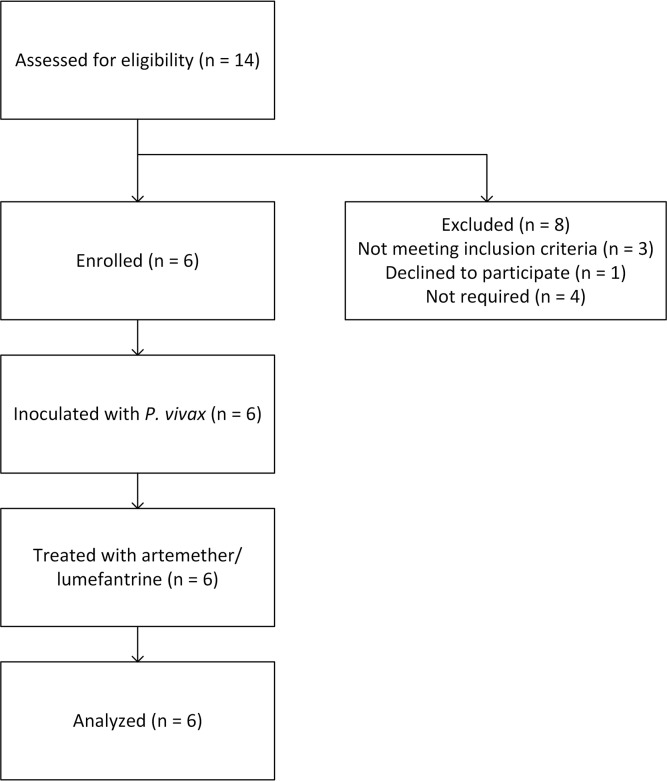
Study flow diagram.

The study was conducted between October 17, 2013 and November 5, 2014. From a total of 14 subjects screened, six subjects were enrolled in this study (Fig 1). Three subjects failed to meet the inclusion/exclusion criteria, one withdrew due to a scheduling conflict and the remaining four eligible study candidates were not required for the study. No enrolled subjects withdrew prior to the completion of the study. The average age of the subjects was 27 years (range 24–32); 50% of the subjects were males. All subjects were of Caucasian origin except for one of Asian origin. Subjects’ mean BMI was 27 kg/m^2^ (range 23.9–29.6 kg/m^2^), mean height was 174 cm (range 163–181 cm), and mean weight was 80 kg (range 72.9–87.1 kg). All subjects completed the study and were included in the outcomes analysis.

### Clinical course

All six subjects were successfully infected with *P*. *vivax*; the clinical time course was consistent across the three cohorts and similar to the previously published pilot study [19]. The mean onset of symptoms was Day 12.2, and ranged from Day 11 (one subject) to Day 13 (two subjects) (S2 Table).

### Safety outcomes

**Fig 2:**
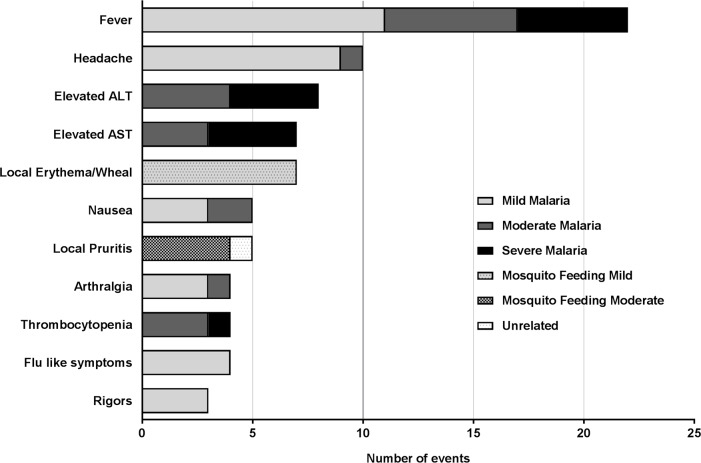
Most frequent adverse events (AEs) reported in the study due to all causes. AE are schematized according to their causality and severity. Fever was the most common AE reported (n = 22). The majority of AEs reported were mild. Only 14 severe AEs were reported and all were attributed to malaria. Abbreviations: ALT: alanine amino transferase; AST: aspartate aminotransferase.

No serious AEs (SAEs) were recorded in any subject during the study. All subjects experienced some symptoms of malaria and these were all outlined in the Participant Informed Consent Form. The total number of AEs reported for the duration of the study was 105, of which the majority (83%; n = 87/105) were attributable to malaria, and 10% (n = 11/105) to direct feeding of mosquitoes (Fig 2). Only one AE was attributed to treatment with A/L; the remaining AEs reported were unrelated to the study treatments (n = 6). Most of the AEs, 57% (n = 60/105), were mild in intensity, with 13% (n = 14/105) severe (S2 Table).

**Fig 3:**
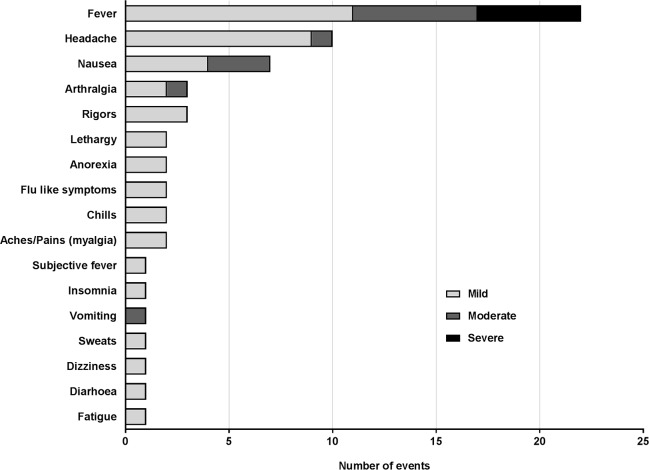
Clinical adverse events attributed to malaria infection by severity. The majority of clinical AEs were mild with only 11 moderate and 5 severe clinical AEs attributable to malaria infection.

The most common AEs reported were fever (n = 22) and headache (n = 10), consistent with malaria infection (Fig 2 and Fig 3). Other clinical AEs reported related to malaria included nausea, arthralgia, chills, flu-like symptoms, rigors, lethargy, anorexia, myalgia, insomnia, vomiting, sweats, dizziness, diarrhea and fatigue (Fig 3).

**Fig 4:**
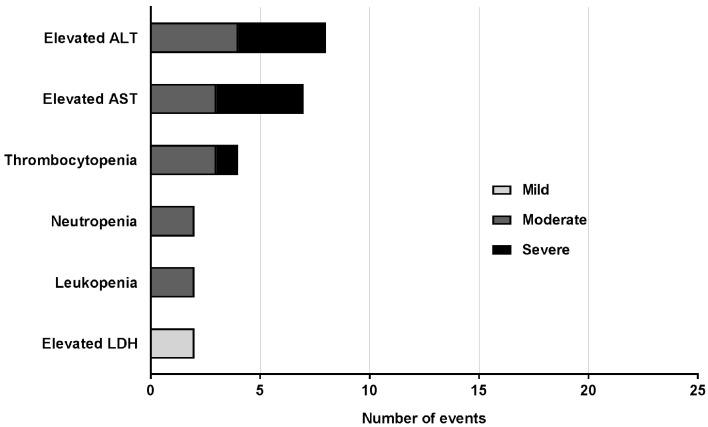
Laboratory parameters recorded as adverse events. All clinically significantly abnormal laboratory parameters are represented according to their severity. All were attributed to malaria infection. There were no significantly abnormal laboratory parameters recorded as AEs attributable to other causes. Of the 25 laboratory parameters that were recorded as AEs, 15 were abnormalities of LFTs, i.e. either an elevation of ALT or AST. The majority of laboratory AEs were moderate in severity with 2 mild and 9 severe. Abbreviations: LDH: lactate dehydrogenase; AST: aspartate aminotransferase; ALT: alanine amino transferase.

The 14 severe AEs recorded were attributed to malaria and included fever ≥39°C (n = 5), one case of transient thrombocytopenia that met the pre-specified criterion of severe (68 x10^9^ platelets/L), four cases of elevated alanine amino transferase (ALT) and four cases of elevated aspartate amino transferase (AST) (Fig 3 and Fig 4). Other laboratory abnormalities, apart from those recorded as AEs of severe intensity were moderate derangements of hematologic parameters, consistent with malaria infection (Fig 4).

Most of the AEs reported (n = 70/105, 67%) were transient in nature and resolved spontaneously without intervention. Twenty-two AEs (21%) resolved following administration of paracetamol. Each subject required at least one dose of paracetamol (S3 Table). Paracetamol use correlated with symptoms of malaria, with two subjects commencing on Day 12, three on Day 13 and one on Day 14. Other than a single dose on Day 20, all subjects ceased paracetamol by Day 16. Subject R003 took the highest number of paracetamol doses, with a total intake of 15 doses taken over a 9-day period. This subject took the recommended maximum allowed daily dose of paracetamol (4 g) on Days 13 and 14. At no stage did any subject receive greater than the recommended maximum dose of 4 g in a 24-hour period.

Anti-emetic medication (ondansetron) was administered to two subjects to treat nausea. One subject received two doses of ondansetron for symptomatic treatment of two separate episodes of moderate nausea. Another subject required both intravenous fluids and anti-emetics (metoclopramide and ondansetron) for moderate nausea. One subject required ranitidine and antacids (magnesium hydroxide, aluminum hydroxide and simethicone) for heartburn of moderate severity, which was attributed to A/L therapy.

### Liver Function Tests

**Fig 5:**
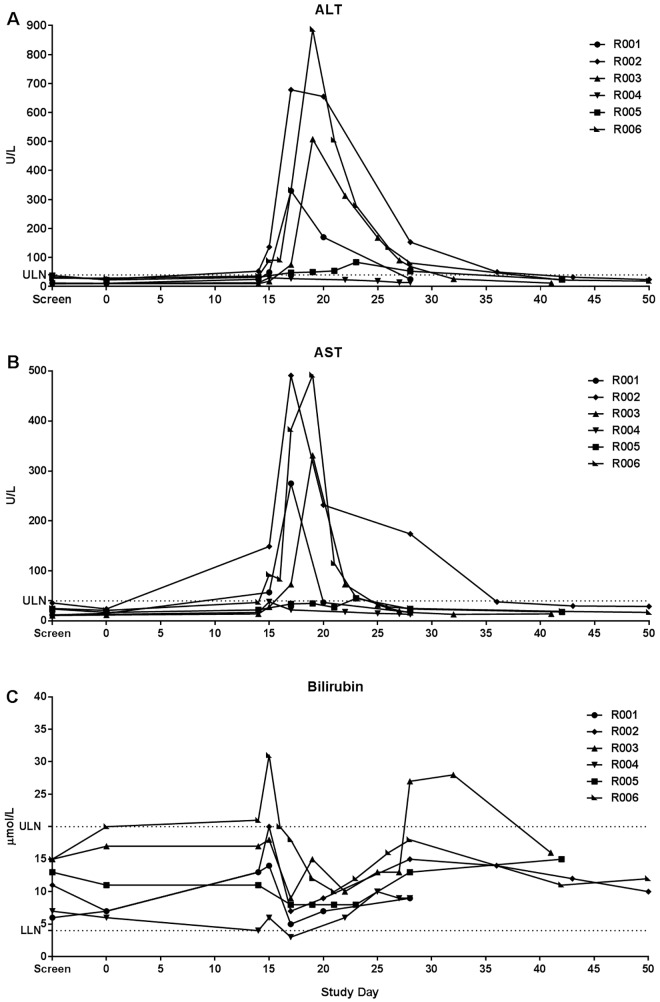
Liver function tests. Levels of (**A**) ALT (alanine aminotransferase), (**B**) AST (aspartate aminotransferase) and (**C**) total bilirubin versus study day for each of the six subjects. The horizontal dotted line indicates normal range. Abbreviations: ULN: upper limit of normal; LLN: lower limit of normal).

Four of the six subjects experienced significant derangements of liver function tests, defined as elevations greater than 5-times the upper limit of normal (ULN), in the form of elevations of both ALT and AST (Fig 5). However, no subject reported any symptoms arising from or accounting for the liver function test derangement. Transaminase elevation was most commonly first detected on Day 15 with an average of Day 16.67 (± 3.2). The highest elevations were seen for Subject R006 on Day 19, with an ALT of 886 U/L (normal range 10–40 U/L) and AST of 492 U/L (normal range 5–40 U/L). ALT peaked most commonly on Day 19, and AST on Day 18. Three of the four subjects who had elevated transaminase levels also had elevations in bilirubin; however, these were mild, transient and did not coincide with the peak transaminase elevation. The highest bilirubin level (31 μmol/L) was observed in Subject R006 on Day 15. As this was less than 2-times the ULN, Hy’s law, used as an indicator for drug-induced liver injury, was not met [31,32]. All liver function tests returned to normal in each subject with no specific intervention. The AST had normalized by Day 27 in most subjects (average Day 27.8, range 17–43) and ALT most commonly by Day 42 (average Day 38.8, range 28–50). Mild and transient elevations of lactate dehydrogenase (LDH) were observed in five of the six subjects, with one subject (R006) having an LDH value deemed clinically significant (730 U/L, normal range 120–250 U/L) on Day 17.

A thorough investigation for the cause of deranged liver function tests was undertaken, including viral serology (HIV, Hepatitis B and C, CMV and EBV, Alphaviruses and Flaviviruses), paracetamol levels, creatinine kinase and liver ultrasound. As the study was conducted in three cohorts of two subjects, following the completion of each cohort a safety review committee meeting was held, and safety findings reviewed. Given the complete spontaneous resolution and lack of concerning features, particularly a significant elevation of bilirubin such that Hy’s law was met, these abnormalities were deemed to not preclude proceeding with subsequent cohorts.

### Tolerability of mosquito feeding

The mosquito feeding was well tolerated, with all subjects completing the scheduled number of direct feeds. Five of the six subjects reported one or more AE resulting from mosquito feeding; however, these were not severe. Of the 11 AEs attributed to mosquito feeding, seven were visible local reactions (wheals or erythema) and were mild in intensity. Four episodes of pruritus were reported, and were described as moderate in intensity. Ten adverse events related to mosquito feeding required intervention in five subjects. Six events in four subjects required both topical (betamethasone dipropionate) and systemic therapy (cetirizine hydrochloride), and four events in two subjects required only topical therapy (one subject required both topical and systemic therapy for one event and topical alone for another two events).

### Inoculum size

The dose of parasites in the inoculum as determined by qPCR of an aliquot of the inoculum was 36,872 parasites for Cohort 1, 18,138 parasites for Cohort 2, and 40,348 parasites for Cohort 3 (mean ±SD: 31,786 ±11,947 parasites). As it was predicted that a significant proportion of parasites would not have survived the freeze-thawing process but would still be detected by qPCR in the inoculum, a linear regression model was developed to estimate the starting dose of viable parasites and the parasite growth rate. We assessed whether there was a significant cohort effect in the growth rate or starting parasite concentration by comparing nested models, and found that the best model contained a cohort effect in the starting inoculum (chi-squared test revealed that the cohort effect provided a significantly better fit, p = 0.017). This observation was in accordance with the qPCR estimation of the inoculum size. The estimated starting concentration of viable parasites was 0.0031 parasites/mL (95% CI: 0.0006–0.0010) for Cohort 1, 0.0050 parasites/mL (95% CI: 0.0007–0.018) for Cohort 2, and 0.0013 parasites/mL (95% CI: 0.0002–0.0047) for Cohort 3 (mean ±SEM: 0.0032 ±0.001 parasites/mL). Assuming an average blood volume of 4,700 mL, the initial infective dose was 15, 24 and 6 parasites for Cohorts 1, 2 and 3, respectively (mean ±SEM: 15 ±5 parasites/mL). Thus, the estimated viability of the *P*. *vivax* inoculum used in this study was 0.01–0.13% (mean ±SEM: 0.06% ±0.04%).

### Parasitemia and parasite multiplication rate (PMR)

**Fig 6:**
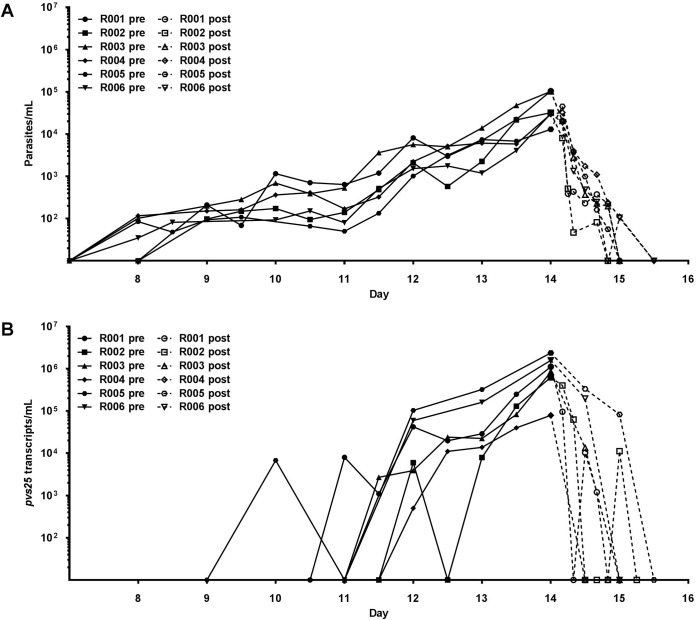
Parasitemia and gametocytemia pre and post antimalarial treatment. (**A**) Parasitemia as determined by qPCR of the 18S rDNA target. (**B**) Estimate of gametocytemia as determined by *pvs25* qRT-PCR. Closed lines or “pre” depicts subjects from first parasitemia detection to treatment with artemether/lumefantrine on Day 14 as per protocol. Dashed lines or “post” represents clearance of the parasitemia following treatment. Day represents study day with inoculation occurring on Day 0.

Very similar parasite kinetics (pre- and post-treatment) were observed across three study cohorts (Fig 6A). Parasites were first detected by PCR in four subjects on Day 8 and in two subjects on Day 9, with parasitemia peaking on Day 14 (the day of antimalarial treatment). The median peak parasitemia detected among study subjects was 31,395 parasites/mL (range 13,045–104,352 parasites/mL). Clearance of parasites after antimalarial treatment was rapid, with all subjects becoming PCR negative by Day 16. We estimated a growth rate of 1.14/day (95% CI: 1.02–1.26) from the mixed effects model fit to the data, which contained a cohort effect on starting parasite concentration. Inclusion of this cohort effect provided a significantly better fit of the data (p = 0.017), using the chi-squared test to compare nested model. This growth rate corresponds to a PMR of 9.9 fold per cycle (95% CI: 7.7–12.4), assuming a 48-hour life cycle for *P*. *vivax*. In a previously reported study [19] the same blood-stage inoculation procedure was used in two subjects belonging to two different cohorts, and who were infected on different days from different cell suspension preparations. When data from these two subjects were incorporated and analyses repeated, the model selection results were unchanged, with the best fitting model including a cohort effect in the starting concentration of parasites but not in parasite growth rate (S1 Supporting Material). These results suggest that from one cohort to the next the growth rate was not significantly different, but that the starting parasite concentration varied. Likewise, when data from all eight subjects was analyzed (six from this study and two from the previous study), the calculated growth rate did not change substantially (1.23/day (95% CI: 1.12–1.35)), corresponding to a PMR of 11.9 fold per cycle (95% CI: 9.3–14.9).

### Course of gametocytemia

Detection of *pvs*25 transcripts as markers of gametocytemia showed the expected kinetics with respect to relationship to asexual parasitemia and emergence of gametocytes. The *pvs*25 level peaked on study Day 14 (median: 490,450 *pvs25* transcripts/mL; range: 39,600–2.3722 x10^6^) immediately prior to the administration of curative antimalarial treatment (Fig 6B). With the exception of Subject R005, who had circulating *pvs*25 levels of 82,900 *pvs25* transcripts/mL, all other subjects demonstrated clearance of gametocytemia by study Day 15 (i.e. 24 hours following administration of the first dose of A/L).

### Transmission to mosquitoes

A total of 16 DFAs (four in Cohort 1; six in Cohort 2; and six in Cohort 3) and 32 MFAs (eight in Cohort 1; 12 in Cohort 2; and 12 in Cohort 3) were conducted in this study. Mosquito feeding began on Day 11 for four subjects (Cohorts 2 and 3) and Day 12 for two subjects (Cohort 1). The average percentage feed success of mosquitoes was 95.6% (±4.9 SD) for DFAs, compared to 92.6% (±7 SD) for MFAs. There was no significant difference in feed success (p = 0.20) between the two feeding methods. Mosquito mortality after feeding was <10% across all cohorts (mean 7% ±6.8; range 0–28%), with a mean mortality rate for mosquitoes fed by DFA of 6.8% (±7.5 SD) compared to 7.2% (±6.3 SD) for mosquitoes fed by MFA.

**Fig 7:**
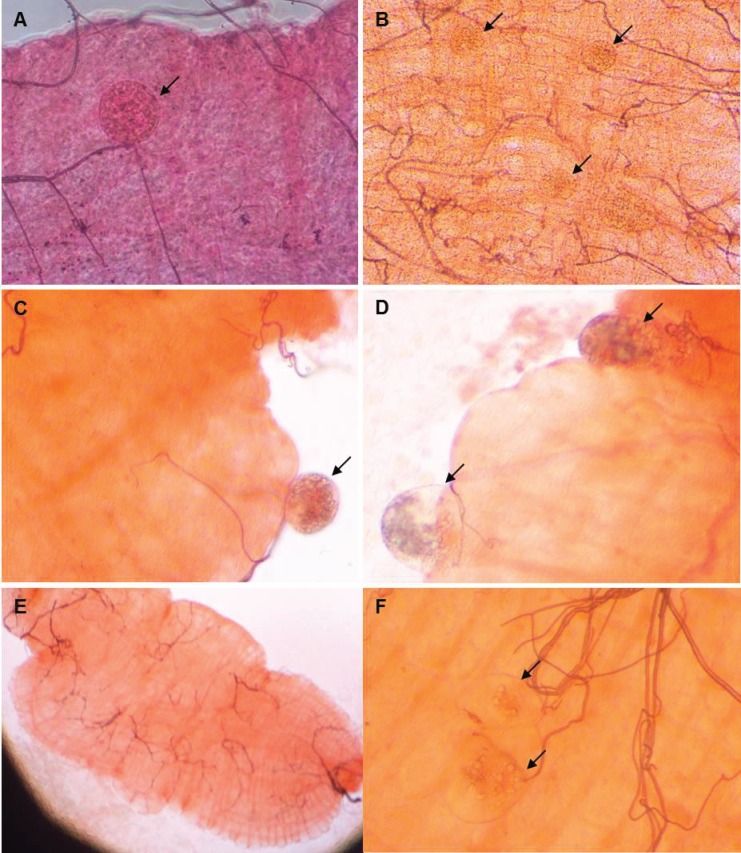
Structures observed on mosquito midguts. **(A)** and **(B)** Oocyts observed on midguts of mosquitoes fed with *P*. *vivax* infected blood. **(C)** and **(D)** Other ovoid structures observed on midguts of mosquitoes fed with *P*. *vivax* infected blood. **(E)** and **(F)** Midguts of mosquitoes fed with non-infected blood (negative control). No structures were present on the mosquito midgut represented in panel E. Panel F illustrates ovoid structures observed on the mosquito midguts of some negative controls. Magnification: 20x in panels A, C, D and F; 10x in panel B; 4x in panel E.

A total of 1801 mosquitoes were dissected for examination of oocysts from 7 to 9 days following blood-feeding (Table 1). In addition, 44 mosquitoes fed by DFA on non-infected blood were dissected (negative control). We detected *Plasmodium* oocysts in 32 dissected mosquitoes, giving an infection prevalence of 1.8% (Fig 7A and 7B). Micrographs of all positive midguts are presented in S2 Supporting Material. Mosquito infectivity was five-times higher for DFA than for MFA, and most of the infections occurred on Day 12 (Table 1).

**Table 1:** Distribution of infected mosquitoes per day of feed in each study cohort according to inoculation method

Cohort	Day of feed	Infection prevalence, % (no. of mosquitoes infected/no. of mosquitoes dissected)		
		DFA	MFA	Total
1	12	NA	0 (0/88)	0 (0/88)
	13	11.6 (7/60)	0 (0/171)	3.0 (7/231)
	14	1.8 (1/56)	1.1 (1/90)	1.4 (2/146)
2	11	NA	2.1 (2/96)	2.1 (2/96)
	12	22.6 (12/53)	2.1 (4 /187)	6.7 (16/240)
	13	0 (0/52)	1.1 (2/179)	0.9 (2/231)
	14	0 (0/55)	1.1 (1/88)	0.7 (1/143)
3	11	NA	0 (0/80)	0 (0/80)
	12	0 (0 /64)	0.6 (1/159)	0.4 (1/223)
	13	0 (0/51)	0.8 (1 /130)	0.6 (1/181)
	14	0 (0/50)	0 (0 /92)	0 (0/142)
**Total**	^**a**^**11(2);12(17):13(10);14(3)**	**4.5 (20/441)**	**0.9 (12/1360)**	**1.8 (32/1801)**

NA, not applicable (no feed performed).

^**a**^The total number of mosquitos infected each feeding day is given in brackets.

During the course of midgut examinations, we frequently encountered other ovoid structures within the midguts of mosquitoes fed on both infected trial subjects and uninfected blood (negative controls). The presence of these structures prompted a detailed morphological analysis of all structures found within midguts of mosquitoes fed on infected blood from a representative set of pictures (n = 599). Key differences in appearance of these structures compared to oocysts included asymmetry, no difference in mercurochrome staining to surrounding mosquito tissue and collapse of the structures upon compression on the coverslip. Apart from the 32 mosquitoes that had structures with the appearance of *Plasmodium* oocysts, morphological analysis of these pictures identified other structures in 523 of the mosquito dissected (87.3%; n = 523/599). These structures were also identified in 28 midguts from negative control mosquitoes (63.6%; n = 28/44).

Molecular and histologic analyses were performed to determine if the structures found in mosquito midguts were associated with fungal or microsporidial, infection. Both, midguts from mosquitos containing atypical structures (n = 4) and whole mosquitoes from the colony (n = 4) tested negative for microsporidia known to infect *A*. *stephensi* (*Nosema* group, *Pleistophora* group and *Brachiola algerae*). There was no amplification of PCR products of the predicted molecular weight with the microsporidia primers sets used. Products outside the predicted molecular weight were confirmed to be non-specific using TA cloning and sequencing. There was no evidence of fungal infection from PAS and Gram chromotrope-stained sections from mosquitoes obtained from the parent colony (n = 20). Ovoid structures within midgut lumens were observed; however, these structures stained similarly to the midgut lining, suggesting that they were of mosquito tissue origin, possibly invaginations of the midgut wall (S3 Supporting Material).

## Discussion

This study demonstrates that our IBSM model using cryobanked *P*. *vivax* parasites is safe and reproducible, and has the potential to test for transmission. This report expands on our previously reported pilot study [19], and increases the total number of subjects inoculated with this *P*. *vivax* malaria parasite bank to eight. The clinical course and parasite growth and clearance were very similar across the three cohorts, and to that reported in the *P*. *vivax* pilot study [19]. This is an important measure of the reproducibility of the model. The use of the highly sensitive qPCR to monitor parasitemia pretreatment as well as the clearance post antimalarial treatment, not only ensures subject safety, but also allows accurate calculation of the parasite growth rate and PMR [33]. Interestingly, there was not a substantial difference in the parasite growth rate and PMR between cohorts, including cohorts conducted in the *P*. *vivax* pilot study, which further supports the reproducibility of the IBSM *P*. *vivax* model. While the ability to benchmark these figures is currently limited owing to a paucity of published data, they are likely to provide a useful tool to compare models and to determine the efficacy of therapeutics and vaccines.

In this study, we report for the first time estimation of parasite viability. The low viability of parasites in the inoculum estimated by linear regression is not surprising given the loss of parasites during the freeze-thaw process. Nevertheless, this does not pose a problem for the IBSM model as the infection rate was 100% and the parasite growth kinetics were consistent between cohorts. We believe that the dose of parasites in the inoculum is more accurately calculated by linear regression than by qPCR, since the latter does not discriminate between viable- and non-viable parasites. Thus, in future IBSM trials we will use the parasite viability estimated by linear regression to calculate the starting parasite concentration.

Although the *P*. *vivax* IBSM system used in this study was safe and well tolerated, liver function test derangements were observed during the study. Including the subjects from the pilot study (n = 2), four out of eight subjects (50%) infected with this *P*. *vivax* inoculum demonstrated significant abnormalities in liver function tests. There was no discernible association with deranged liver function tests and any subject factors including age, sex, BMI, or gender. One subject (R002) had a minor derangement of both ALT and AST at baseline (ALT 36 U/L, normal range 10–35; AST 32 U/L, normal range 5–30). This subject had the second greatest deviation in LFT’s (peak ALT 678 U/L, AST 491 U/L), potentially indicative of an underlying predisposition, and highlights the requirement to ideally enroll subjects with baseline LFTs all within the normal range.

Liver function test derangements have been well recognized for a long time to be associated with *P*. *vivax* infection [34]. Prior to modern liver function testing, observations consistent with hepatic dysfunction including protein derangements and abnormal cephalin flocculation tests were observed during malaria-therapy for syphilis [35–37]. A study by McMahon and colleagues [38] showed that all 52 patients infected with *P*. *vivax* in whom liver function testing was performed had detectable abnormalities of liver function. In this study, liver biopsies were performed in 62% of the cases yielding abnormalities in 91.9% of those patients. Based on the timing and pattern of both the liver function test profiles and histological changes they reported it was very improbable that either of these abnormalities could have been caused by the antimalarial therapy administered. In fact, significant improvements were observed in response to treatment. More recently, a number of studies conducted in India have provided further evidence for hepatic dysfunction arising in response to malaria infection [39–44]. While the exact mechanism underlying LFT derangement remains to be elucidated, some clues can be deduced from previous studies. In children dying with *P*. *falciparum* malaria in Malawi, hemozoin-filled Kupffer cells were invariably present and thought to be the source of inflammatory signals [45]. Mouse studies suggest that hemazoin-induced inflammation is responsible for hepatic dysfunction [46], while other studies suggest that mitochondrial pathology and oxidative stress promote hepatocyte apoptosis [47]. Of note, the donor of the *P*. *vivax* inoculum (who acquired malaria in the Solomon Islands) also demonstrated abnormal liver function tests with a peak AST of 362 U/L (normal range <31) and ALT of 197 U/L (normal range <34).

Other studies have suggested that the regular ingestion of the maximum daily dose of 4 g of paracetamol for 14 days is sufficient to induce a significant increase in median maximum ALT by a factor of 2.78 compared with placebo [48]. In the study conducted by Watkins and colleagues, elevations of ALT to more than 3 times the ULN occurred after at least eight doses of paracetamol had been administered during a 3-day period. In the present study, the greatest number of doses of paracetamol administered was 15 over a 9-day period. Subject R003, who ingested the highest cumulative dose of paracetamol (15 doses), had the third highest ALT and AST peak. The subject with the greatest LFT abnormality, R006, received a total of five doses of paracetamol over a 3-day period. The peak of LFT derangements was observed approximately 3 to 4 days after all but one subject ceased paracetamol. This is consistent with the study conducted by Watkins and colleagues, in which the ALT continued to increase up to 4 days after ceasing paracetamol treatment [48]. An alternative antipyretic such as ibuprofen could be used in subsequent studies to investigate if this reduces the incidence of LFT abnormalities. Published studies suggest that ibuprofen is at least as good as paracetamol for relief of symptoms of malaria [49].

The relatively high rate of liver function test abnormalities is likely multifactorial, driven mostly by malaria infection itself and possibly contributed to by the administration of paracetamol and a predisposition in some subjects. Clearly, it warrants close observation and further investigation. However, given the transient nature, spontaneous resolution and lack of symptoms associated with liver derangements in infected subjects, these laboratory findings should not preclude future studies utilizing this *P*. *vivax* HMPBS-Pv malaria cell bank.

Experiments testing transmission of *Plasmodium* to mosquitoes via DFA and MFA were successful and well tolerated. Oocysts were observed on the midguts of infected mosquitoes, as confirmed by author JS, an expert in mosquito midgut observation using micrographs. Other structures were also observed on mosquito midguts; however, there were critical differences on the morphology of these structures compared to *Plasmodium* oocysts. Although we could not determine the identity of these structures, the need to distinguish oocysts from similar appearing structures (including mosquito adipose cells, hemocytes and out-pocketings of the midgut epithelium) has been previously noted [50]. Uncertainty regarding classification of structures present in infected mosquito midguts is of concern if the system is to be used to assess clinical endpoints such as efficacy of a transmission-blocking vaccine. Therefore, in future studies verification methods for oocyst identification such as immunofluorescence assay or PCR should be conducted.

The prevalence of mosquito infection achieved in this study was very low. Other *P*. *vivax* transmission studies have reported infection prevalence levels >50%, both by DFA [51] and MFA [52]. We observed higher mosquito infectivity from DFA than MFA, in accordance with the literature in field transmission studies [52–54]. We are not certain why the prevalence of mosquito infection was so low. Successful establishment of infection to mosquito vectors can be influenced by a multitude of factors. We do not rule out the possibility of parasite vector incompatibility in this study, as the parasite inoculum originated from a parasitemic donor who had come from the Solomon Islands, and the laboratory colony of *A*. *stephensi* at QIMR Berghofer originally came from India. The vector species used in this IBSM model, *A*. *stephensi*, is an efficient vector of *P*. *falciparum* [23,55,56] and *P*. *vivax* [23,57]. Geographical compatibility of parasite and vector has yet to be established in the context of *P*. *vivax* transmission studies using IBSM. To the best of our knowledge, there are no data describing the susceptibility of *A*. *stephensi* to *P*. *vivax* infection in the context of IBSM. The susceptibility of the *A*. *stephensi* vector to infection by *P*. *vivax* parasites from the bank used in this study has not been validated owing to the inability to culture parasites of this species *in vitro*.

Although the probability of mosquito infection increases with increasing gametocyte density, several studies suggest that transmission of *P*. *vivax* may occur when gametocytemia is submicroscopic [58–61]. Recently, Vallejo and colleagues [62] failed to infect mosquitoes fed on blood from subjects experimentally infected with *P*. *vivax* sporozoites. Transmission to mosquitoes was not achieved even though high levels of gametocytes were present in subjects’ blood at the time of mosquito feeding. Other factors such as quality or maturity of both male and female gametocytes present in infected blood of the subjects are also expected to play a significant role in transmission success. In this study, gametocytemia was detected by the marker *pvs25*. Unfortunately, the number of *pvs25* transcripts per gametocyte is not currently known, and in the absence of a suitable reference standard it is not possible to make this assay quantitative. Given *pvs25* is likely female specific [63], development of a method that detects both male and female gametocytes would be useful to better understand success of malaria transmission to mosquito vectors. More detailed assessment of the development of gametocytes would be facilitated by similar assays detecting other markers of gametocyte development such as alpha tubulin II, pvs48/45 and *pvg377* [64–66].

In conclusion, herein we report a safe and reproducible IBSM challenge system utilizing cryobanked *P*. *vivax*. This *P*. *vivax* IBSM model promises to be a useful tool to expedite the assessment of therapeutic interventions. Moreover, the addition of mosquito transmission experiments represents a potential model for the evaluation of transmission blocking interventions.

## Supporting Information

S1 Supporting Information CONSORT checklist (PDF)

S2 Supporting Information Full inclusion and exclusion criteria (PDF)

S1 Table Schedule of events (PDF)

S2 Table Symptoms and adverse events (PDF)

S3 Table Paracetamol doses by subject and study day (PDF)

S1 Supporting Material Comparison of estimates of inoculum size, growth rate and PMR with a previously published IBSM *P*. *vivax* study (PDF)

S2 Supporting Material Micrograph images of oocysts and other ovoid structures present in mosquito midguts (PDF)

S3 Supporting Material Periodic Acid Schiff (PAS) Gram chromotrope stained sections of non-*Plasmodium* infected *Anopheles stephensi* (PDF)
