# Supplementary material for: Safety and Reproducibility of a Clinical Trial System Using Induced Blood Stage Plasmodium vivax Infection and Its Potential as a Model to Evaluate Malaria Transmission
Source: PLoS Negl Trop Dis. 2016 Dec 8;10(12):e0005139. doi: 10.1371/journal.pntd.0005139 (PMC5145139; doi:10.1371/journal.pntd.0005139)
Supplement: S2 Supporting Information — (PDF) [file pntd.0005139.s002.pdf]

S2 Supporting Information. Full Inclusion and Exclusion Criteria

Inclusion criteria

1. Adults (males and non pregnant and non lactating females), aged between 18 and 50 years who do not live alone (from Day 0 until at least the end of the antimalarial drug treatment).
2. A BMI within the range 18–30 kg/m^2 and must weigh more than 50kg.
3. Participants must understand the procedures involved and agree to participate in the study by giving fully informed, written consent.
4. Be contactable and available for the duration of the trial and be available up to 2 weeks following end of study visit. (maximum of 6 weeks in total from time of malaria challenge), and reachable (24/7) by mobile phone during the period between malaria challenge and completion of antimalarial treatment).
5. Able and willing (in the Investigator's opinion) to comply with all study requirements.
6. Participants must be non-smokers for at least three months prior to screening and in good health, as assessed during pre-study medical examination and by review of screening results. (Note: "Tobacco use" includes smoking and the use of snuff and chewing tobacco, and other nicotine or nicotine containing products). Social smokers who smoke up to 10 cigarettes per month may be allowed to participate at the investigators discretion.
7. Female participants of childbearing potential must have adequate contraception in place (see section 5.11) for the duration of the study, and have negative results on a serum or urine pregnancy test done before administration of study product. Note: Adequate contraception includes surgical sterility by irreversible bilateral oophorectomy or bilateral salpingectomy (but not tubal ligation) (with or without hysterectomy) at least 6 months ago, or use of an insertable, injectable, transdermal, or combination oral contraceptive approved by the US FDA or TGA combined with a barrier contraceptive (female condom

or male partners to use condom) through completion of the study and have negative results on a serum or urine pregnancy test done before administration of study medication.

8. Good peripheral venous access.
9. Blood group A Duffy antigen positive.

Exclusion Criteria

Participants may be excluded from the study either during screening, on blood stage challenge days, or during the blood sampling intervals, for any of the following reasons:

1. History of clinical malaria (any species).
2. Travelled to or lived (>2 weeks) in a malaria-endemic country during the past 12 months or planning travel to a malaria-endemic country during the course of the study.
3. Known severe reaction to mosquito bites other than local itching and redness
4. Evidence of increased cardiovascular disease risk (defined as >10%, 5 year risk) as determined by the method of Gaziano et al. [1]. Risk factors include sex, age, systolic blood pressure (mm Hg), smoking status, body mass index (BMI, kg/m^2), reported diabetes status and blood pressure.
5. History of splenectomy.
6. Pregnant or breast feeding (all women must have a negative pregnancy test result prior to study product administration).
7. History of a severe allergic reaction, anaphylaxis or convulsions following any vaccination or infusion.
8. Presence of current or suspected serious chronic diseases such as cardiac or autoimmune disease (HIV or other immunodeficiencies), insulin dependent diabetes, progressive neurological disease, severe malnutrition, acute or progressive hepatic disease, acute or

progressive renal disease, psoriasis, rheumatoid arthritis, asthma, epilepsy or obsessive compulsive disorder, skin carcinoma excluding non-spreadable skin cancers such as basal cell and squamous cell carcinoma, schizophrenia, bi-polar disease, or other severe (disabling) chronic psychiatric diagnosis.

9. History of sickle cell anemia, sickle cell trait, thalassemia or thalassemia trait, or any hematological condition that could affect susceptibility to malaria infection.
10. The participant is receiving psychiatric drugs[1] or has been hospitalized within the past 5 years prior to enrollment for psychiatric illness, history of suicide attempt or confinement for danger to self or others. Participants who are receiving a single antidepressant drug and are stable for at least 3 months prior to enrollment without decompensating may be allowed to enroll in the study at the investigator's discretion.
11. Known inherited genetic anomaly (known as cytogenetic disorders) e.g., Down's syndrome.
12. Participants unwilling to defer blood donations for the duration of the trial and for at least 6 months after the end of their involvement in the study.
13. A history of clinically significant ECG abnormalities and known pre-existing prolongation of the QTc interval considered clinically significant.
14. Family history of congenital prolongation of the QTc interval on electrocardiograms or of sudden death or any other clinical condition known to prolong the QTcB interval, e.g. participants with a history of symptomatic cardiac arrhythmias, with clinically relevant bradycardia or with severe cardiac disease;
15. ECG findings:
    a. Electrocardiogram (ECG) abnormalities in the standard 12-lead ECG (at screening) which in the opinion of the Investigator is clinically relevant or will interfere with the ECG analysis.

b. Any of the following ECG abnormalities at screening or on admission:

   i. PR >210 msec

   ii. QRS complex >110 msec

   iii. QTcB or QTcF > 450 msec for males or > 470 msec for females or shortened QTcF < 340 msec

   iv. Any degree of heart block

16. Recent or current therapy (within 30 days of malaria challenge) with an antibiotic or drug with potential antimalarial activity (tetracycline, azithromycin, clindamycin, hydroxychloroquine etc.).

17. Known hypersensitivity to artemether or lumefantrine or any other known contraindications to the use of Riamet and the alternative anti-malarial medication, Malarone.

18. Concomitant use of any drug which is metabolized by the cytochrome enzyme CYP2D6 (e.g. flecainide, metoprolol, imipramine, amitriptyline, clomipramine) OR drugs that are known to prolong the QTcB interval, e.g. antiarrhythmics of classes IA and III, neuroleptics, antidepressant agents, certain antibiotics (including some agents of the following classes: macrolides, fluoroquinolones, imidazole and triazole antifungal agents), certain nonsedating antihistamines (terfenadine, astemizole), cisapride. Use moderate or strong inhibitors and/or inducers of CYP450 within 4 weeks prior to the planned first drug administration (or at least 5 half-lives of the compound whichever period is the longer).

19. Use of corticosteroids, anti-inflammatory drugs, any immunomodulators or anticoagulants. Currently receiving or have previously received immunosuppressive therapy, including systemic steroids including ACTH or inhaled steroids in dosages which are associated with hypothalamic-pituitary-adrenal axis suppression such as

1mg/kg/day of prednisone or its equivalent or chronic use of inhaled high potency corticosteroids (budesonide 800 μg per day or fluticasone 750 μg).

20. Presence of acute infectious disease or fever (e.g., sub-lingual temperature ≥ 38.5°C) within the five days prior to study product administration.
21. Evidence of acute illness within the four weeks before trial prior to screening.
22. Significant intercurrent disease of any type, in particular liver, renal, cardiac, pulmonary, neurologic, rheumatologic, or autoimmune disease by history, physical examination, and/or laboratory studies including urinalysis.
23. Alcohol consumption greater than community norms (i.e. more than 21 standard drinks per week for males and 14 standard drinks per week for females).
24. A history of drug habituation, or any prior intravenous usage of an illicit substance.
25. Medical requirement for intravenous immunoglobulin or blood transfusions.
26. Participation in any investigational product study within the 8 weeks preceding the study.
27. Participation in any research study involving significant blood sampling, or blood donation to Red Cross (or other) blood bank during the 8 weeks preceding the rescue drug dose in the study.
28. Prior receipt of an investigational malaria vaccine or any other investigational vaccine likely to impact on interpretation of the trial data.
29. Have ever received a blood transfusion.
30. Positive test for HIV, Hepatitis B or Hepatitis C.
31. Any clinically significant biochemical or hematologic abnormality (Hb must be ≥11.5g/dL for females; 13.5g/dL for males) – including pre-existing red cell antibodies. Electrolyte disturbances, particularly hypokalemia, hypocalcaemia or hypomagnesaemia.
32. Participation in unaccustomed strenuous exercise within 7 days prior to Screening or any study visit.

33. Ingestion of any poppy seeds within the 24 hours prior to the screening blood test (participants will be advised by phone not to consume any poppy seed in this time period).
34. Detection of any drug listed in table 2 in the urine drug screen unless there is an explanation acceptable to the medical investigator (e.g. the participant has stated in advance that they consumed a prescription or OTC product which contained the detected drug) and/or the participant has a negative urine drug screen on retest by the pathology laboratory.
35. Evidence of any condition that, in the opinion of the clinical investigator, might interfere with the evaluation of the study objectives or pose excessive risks to participants.
36. At the discretion of the Investigator the use of prescription or OTC medications, within 2 weeks of BSPC administration, or within 2 weeks of administration of the antimalarial drug (or at least 5 half-lives of the compound whichever period is the longer). Excluded from this list is intermittent use of paracetamol at doses of <2 g/day and oral contraceptives (combination estrogen/progesterone pills), injectable progesterone or subdermal implants.

References

1. Gaziano TA, Young CR, Fitzmaurice G, Atwood S, Gaziano JM (2008) Laboratory-based versus non-laboratory-based method for assessment of cardiovascular disease risk: the NHANES I Follow-up Study cohort. Lancet 371: 923-931.
