# Supplementary material for: Safety and Reproducibility of a Clinical Trial System Using Induced Blood Stage Plasmodium vivax Infection and Its Potential as a Model to Evaluate Malaria Transmission
Source: PLoS Negl Trop Dis. 2016 Dec 8;10(12):e0005139. doi: 10.1371/journal.pntd.0005139 (PMC5145139; doi:10.1371/journal.pntd.0005139)
Supplement: S1 Table — (PDF) [file pntd.0005139.s003.pdf]

S1 Table. Schedule of Events

**Procedures**	**Screen**	**Challenge inoculum**		**Malaria monitoring**	**Mosquito feeding days**	**Drug treatment**			**Safety monitoring**	**Follow up visit**
**Day**	**~D28 to –D2**	**Day 0**	**D1 to D6**	**From D7AM only until PCR positive and then AM&PM until mosquitoes feeding day**	**~ D 11, 12, 13, 14**	**Admission ~D14**	**Confinement at clinical site ~D14-15AM**	**Exit of Unit ~D15PM**	**Up to 2 days post treatment**	**D28±2**
Informed consent & eligibility	x	x								
Medical history	x	x								x
Physical examination [a]	x	x		x	x	x	x	x	x	x
ECG [b]	x									x
Vital signs [c] – Temperature, HR, RR, BP	x	x		x	x	x	x	x	x	x
Hematology & biochemistry [d]	x	x				x			x	x
Serology [e]	x									x
Red cell alloantibody	x									x
Urinalysis/pregnancy for female participants [f]	x	x				x				x
Urine drug screen & alcohol breath test [g]	x	x				x				
**Blood stage challenge**		x								
**Phone call**			x							
Mosquitoes transmission [k]					x					
Blood collection for *P.vivax* infection monitoring		x			x	x		x		x
Unit confinement [h]						x	x			
**Drug treatment**							x[i]	x[i]	x[i]	
Malaria qRT-PCR [j] (thick films)				x	x (including thick films)	x	x	x	x	x
Safety serum storage		x								x

[a] A complete physical examination was to be performed at screening and D28 or EOS. An abbreviated physical examination was to be performed upon admission to the clinical unit and at all morning and evening visits during confinement and follow up visits and where symptoms of malaria were identified.

[b] ECG – 12-lead electrocardiogram was to be recorded once at screening, and at D28 or EOS.

[c] Temperature (sub-lingual), RR, HR and BP were to be obtained at screening, pre-dose malaria inoculum, and at approximately 60 min post inoculum/ prior to leaving the unit and D28 (EOS). Vital signs were to be measured also at each outpatient visit and at morning, midday and evening during confinement or when malaria symptoms were present. Subjects were to remain semirecumbent for 5 minutes prior to measurement of HR and BP.

[d] Hematology and chemistry tests were to be performed at screening, D0 pre-malaria inoculum, admission to the clinical research unit and dosing of antimalarial agent, D17 AM and D28 or EOS. Reticulocytes were to be assessed at D0 baseline and D28 visit or EOS. Reticulocytes were to be assessed at D0 (baseline) and D28 or EOS. Liver function was to be monitored as on an outpatient basis 2-3 times per week following the EOS visit as advised by the Medical Investigator.

[e] HIV, Hepatitis B and Hepatitis C were to be tested at screening only. EBV and CMV were to be tested at screening and at EOS.

[f] Serum B-HCG was to be tested at screening; urine B-HCG was to be tested at D0, on admission for Riamet® treatment to the unit (D14AM) and at D28 or EOS.

[g] Drug screen and alcohol breath test were to tested at screening, D0 pre-inoculum and at entry into clinical unit (D14 AM or as advised by the Medical Investigator).

[h] Confinement period of 36 hours (from D14 AM to D15PM) or as advised by the clinical staff.

[i] Drug treatment from first clinical symptoms of malaria infection for required period of drug treatment, i.e. 3 days or varied per drug schedule in protocol.

[j] qRT-PCR was to be performed at D0 (baseline); from D7 AM only until PCR positive for malaria, then AM and PM until mosquito feeding day; AM-only blood samples were be collected for PCR analysis during mosquito feeding days. During confinement, samples for PCR were to be collected every 6 hours between D14 AM and D15 PM (i.e. for 36 hours after initiation of treatment). PCR blood samples were to be collected AM and PM on D16 following discharge from the clinical unit and them AM only on D17 and at the final visit (Day 28/EOS). Malaria thick films were to be prepared from blood samples collected from Day 11, 12, 13 and D14 (mosquitoes feeding days).

[k] Malaria transmission experiments were to be undertaken on the 3 days leading up to the anticipated commencement of treatment or onset of malaria symptoms (~D11, D12, D13 and D14). For MFAs with *A. stephensi*, 10 mL of blood were to be collected from each participant AM and PM into heparinized vacutainer tubes. To prevent premature exflagellation, blood was to be kept and transferred at 38°C (within 5 minutes of collection) until dispensed into membrane feeders. For DFAs, subjects were to visit the quarantine insectary facility at QIMR Berghofer, where they were to allow vector mosquitoes to feed on alternating sites of the volar surface of their forearms or thighs for a period of 15 ±5 minutes. Experimental infection of mosquitoes by DFA and MFA was to be repeated 2-3 times until subjects commenced antimalarial treatment.

Abbreviations: **D**, day; **HR**, heart rate, **RR**, respiratory rate; **BP**, blood pressure; **qRT-PCR,** quantitative reverse transcriptase PCR; **EOS**, End of Study; **DFA**, direct feeding assay; **MFA**, membrane feeding assay.
