# Supplementary material for: Safety and Reproducibility of a Clinical Trial System Using Induced Blood Stage Plasmodium vivax Infection and Its Potential as a Model to Evaluate Malaria Transmission
Source: PLoS Negl Trop Dis. 2016 Dec 8;10(12):e0005139. doi: 10.1371/journal.pntd.0005139 (PMC5145139; doi:10.1371/journal.pntd.0005139)
Supplement: S2 Table — (PDF) [file pntd.0005139.s004.pdf]

S2 Table. Symptoms and Adverse Events (AEs)

Subject	Symptom onset (day)	Symptom duration post treatment (hrs)	Total AEs	Mild AEs	Moderate AEs	Severe AEs	AEs attributed to inoculum	AEs attributed to mosquitoes	AEs attributed to A/L	Unrelated AEs
**R001**	11	24	18	11	5	2	13	3	1	1
**R002**	12	24	23	10	11	2	18	2	0	3
**R003**	12	12	20	11	5	6	18	3	0	1
**R004**	12	24	9	7	1	0	6	1	0	1
**R005**	13	48	12	7	4	1	12	0	0	0
**R006**	13	36	17	14	5	3	20	2	0	0
**Average**	12.2	28.0	16.5	10.0	5.2	2.3	14.5	1.8	0.2	1.0
**Total**			99	60	31	14	87	11	1	6

Subjects were inoculated on Day 0 and treated with artemether/lumefantrine (A/L) as per protocol on Day 14. No serious AEs were recorded for any subject. AEs attributed to inoculum refers to AEs consistent with malaria infection (i.e. the inoculum). AEs attributed to mosquitoes refers to AEs attributed to bites from mosquitoes during direct mosquito feeding assays (DFA). AEs were deemed unrelated if not relating to either malaria infection, mosquito feeding or antimalarial treatment.
