# Supplementary material for: Safety and Reproducibility of a Clinical Trial System Using Induced Blood Stage Plasmodium vivax Infection and Its Potential as a Model to Evaluate Malaria Transmission
Source: PLoS Negl Trop Dis. 2016 Dec 8;10(12):e0005139. doi: 10.1371/journal.pntd.0005139 (PMC5145139; doi:10.1371/journal.pntd.0005139)
Supplement: S3 Table — (PDF) [file pntd.0005139.s005.pdf]

S3 Table. Paracetamol doses by subject and study day

Subject	Study day	Paracetamol doses (1 g/dose)	Total paracetamol doses taken during study
**R001**	13	2	**8**
	14	3	
	15	3	
**R002**	12	1	**10**
	13	4	
	14	4	
	15	1	
**R003**	12	1	**15**
	13	4	
	14	4	
	15	2	
	16	3	
	20	1	
**R004**	14	1	**2**
	15	1	
**R005**	13	1	**5**
	14	2	
	15	2	
**R006**	13	1	**5**
	14	3	
	15	1	
