# Supplementary material for: Safety and Reproducibility of a Clinical Trial System Using Induced Blood Stage Plasmodium vivax Infection and Its Potential as a Model to Evaluate Malaria Transmission
Source: PLoS Negl Trop Dis. 2016 Dec 8;10(12):e0005139. doi: 10.1371/journal.pntd.0005139 (PMC5145139; doi:10.1371/journal.pntd.0005139)
Supplement: S1 Supporting Material — (PDF) [file pntd.0005139.s006.pdf]

S1 Supporting Material. Comparison of estimates of inoculum size, growth rate and PMR with a previously published IBSM *P. vivax* study

Methods

A linear mixed effects model was fitted to the log-transformed parasitemia data for two subjects who belonged to two different cohorts of a previously reported study [1], referred to here as “pilot study”. In the pilot study the same blood-stage inoculation procedure was used as in the present study. Data from the two cohorts of the pilot study were compared with the three cohorts presented in this paper. A linear mixed effects model was also fitted to all eight subjects (six from this study and two from the pilot study). We assessed whether there were any significant cohort effects on the growth rate or the y-intercept after the inclusion of these two subjects in the analysis, and selected the best model using backwards elimination with the chi-squared test, and log-likelihood of fitted models with the same number of parameters.

Results

We estimated a growth rate of 1.52/day (95% CI: 1.24-1.79) for the two subjects in the pilot study, corresponding to a PMR of 21.5 (95% CI: 11.9-35.9). The average starting concentration was estimated to be $8x10^{-5}$ parasites/mL (95% CI: $9x10^{-7}$ - $5x10^{-4}$).

We then analyzed together the two cohorts from the pilot study and the three cohorts from the present study and re-assessed for any cohort effects on the growth rate or y-intercept, observing that the result were unchanged. The best model to explain the data has a cohort effect in the starting concentration of parasites but not in the growth rate. These results suggest that from one cohort to the next the growth rate was not significantly different in these trials, but that the starting inoculum varied. When fitting this model with a cohort effect in the starting concentration of parasites to all eight subjects we obtained a new estimate of the growth rate (1.23/day [95% CI: 1.12-1.35]), corresponding to a PMR of 11.9 (95% CI:

9.3-14.9). Further, the starting parasite concentrations for each cohort were estimated to be 0.0011 (95% CI: 0.0002-0.0036), 0.0018 (95% CI: 0.0003-0.0062), 0.0005 (95% CI: 0.0001-0.0017) parasites/mL for Cohorts 1, 2 and 3 of this study respectively, and 0.0007 (95% CI: 0.0001-0.0028) and 0.0008 (95% CI: 0.0001-0.0028) parasites/mL for Cohorts 1 and 2 of the pilot study.

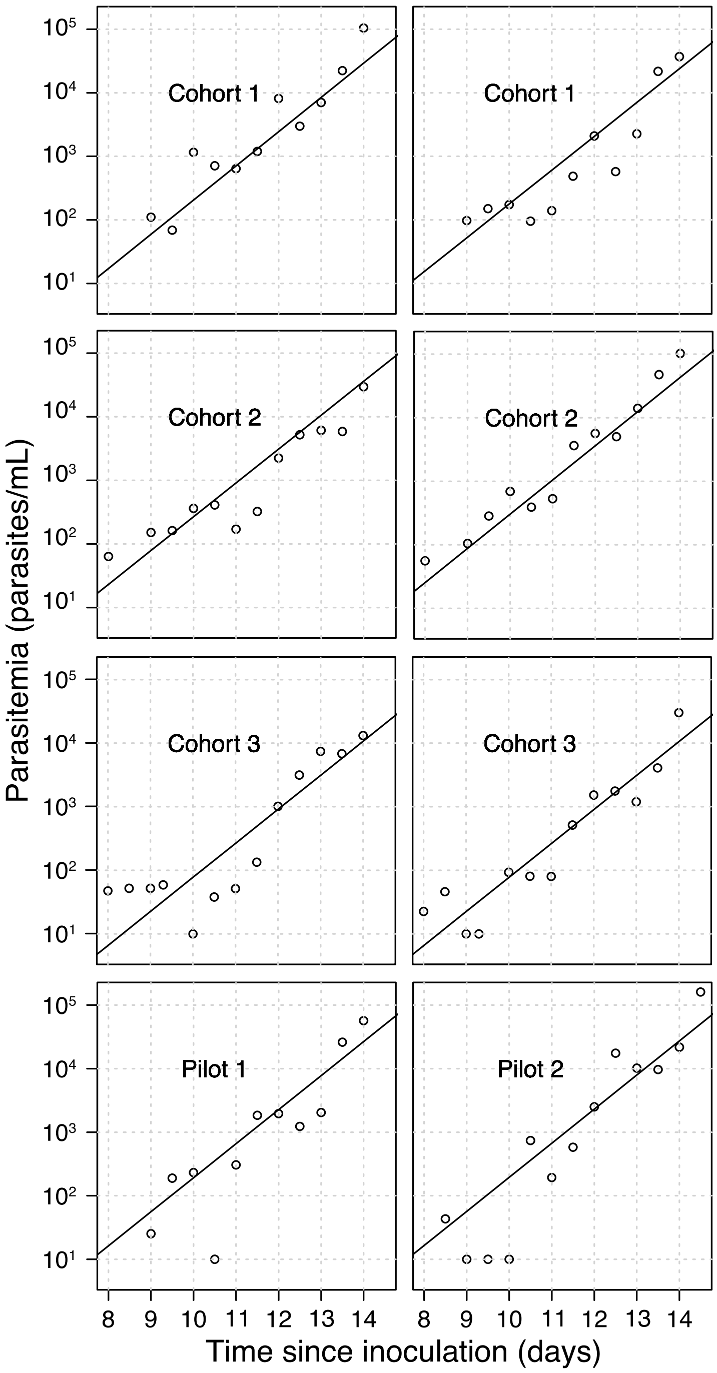

Figure 1. Fitted mixed effects model of parasite growth in eight subjects infected with IBSM *P. vivax*. Cohorts 1-3 of the present study, and subjects 1 and 2 from the pilot study are shown. Circles indicate the parasitemia measured before antimalarial treatment by qPCR. The line indicates the fitted mixed effects model of parasite growth with the cohort effect in the starting parasite concentration. The PMR is equivalent in all cohorts, but the starting inoculum varies between cohorts.

References

1. McCarthy JS, Griffin PM, Sekuloski S, Bright AT, Rockett R, Looke D, et al. (2013). Experimentally induced blood-stage *Plasmodium vivax* infection in healthy volunteers. J Infect Dis 208: 1688-1694.
