# Supplementary material for: Safety and Reproducibility of a Clinical Trial System Using Induced Blood Stage Plasmodium vivax Infection and Its Potential as a Model to Evaluate Malaria Transmission
Source: PLoS Negl Trop Dis. 2016 Dec 8;10(12):e0005139. doi: 10.1371/journal.pntd.0005139 (PMC5145139; doi:10.1371/journal.pntd.0005139)
Supplement: S2 Supporting Material — (PDF) [file pntd.0005139.s007.pdf]

S2 Supporting Material. Micrograph images of oocysts and other ovoid structures present in mosquito midguts. (A) Micrograph images of 32 mosquito midguts infected with *P. vivax*. Oocysts are indicated with arrows. (**B**) Representative micrograph images of other ovoid structures observed on mosquito midguts fed with *P. vivax* infected blood (panels 1-16) and non-infected blood (panels 17-24, control mosquitoes). Ovoid structures are indicated with arrows.

(A) Micrograph images of 32 mosquito midguts infected with *P. vivax*

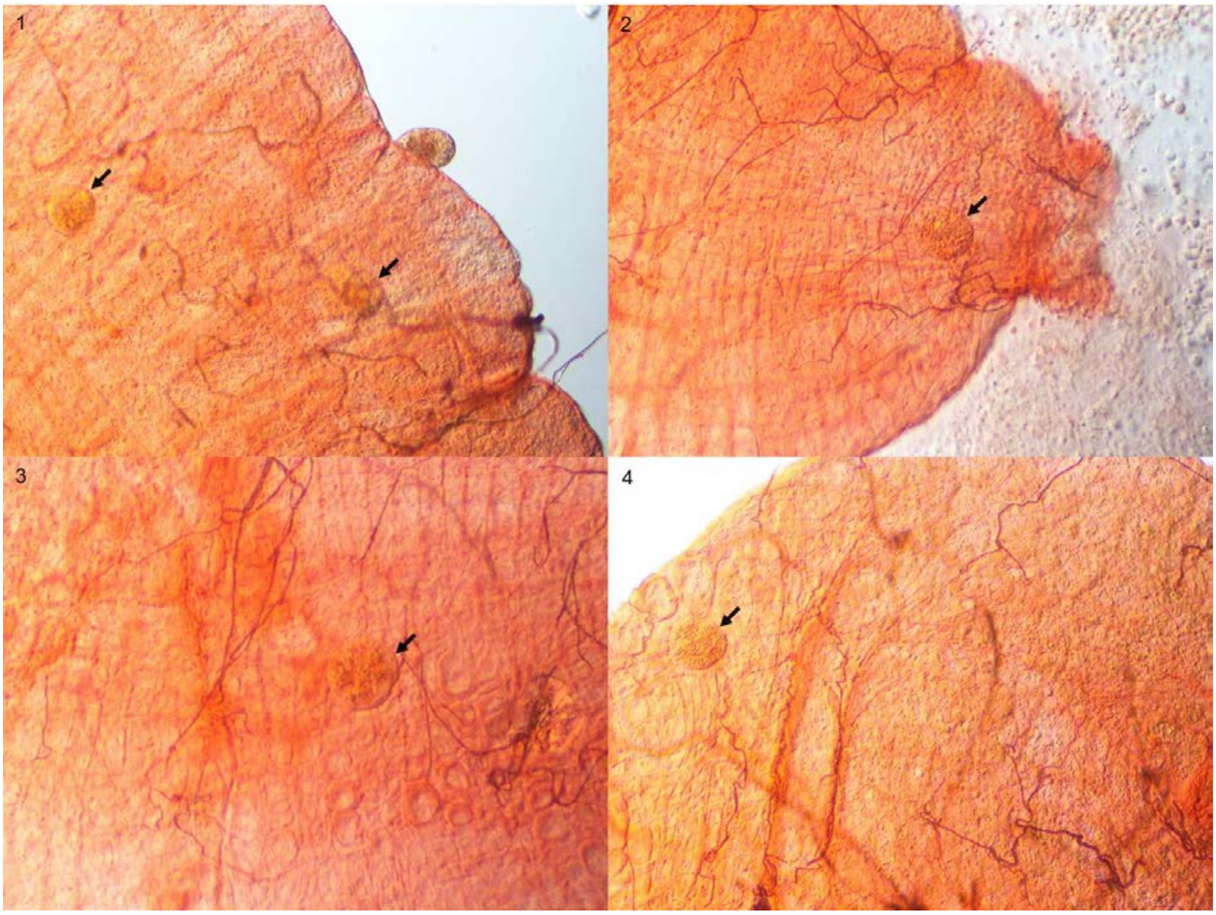

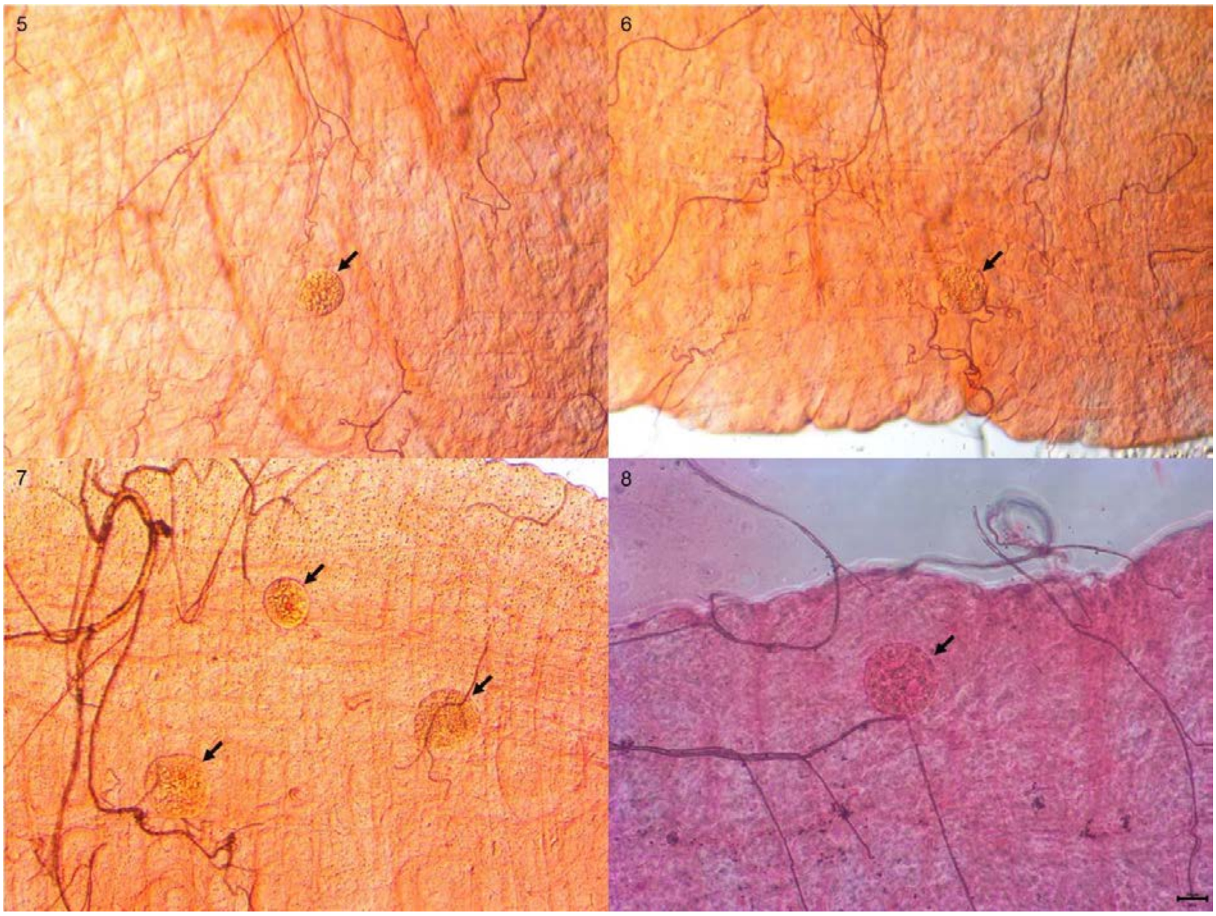
5
6
7
8

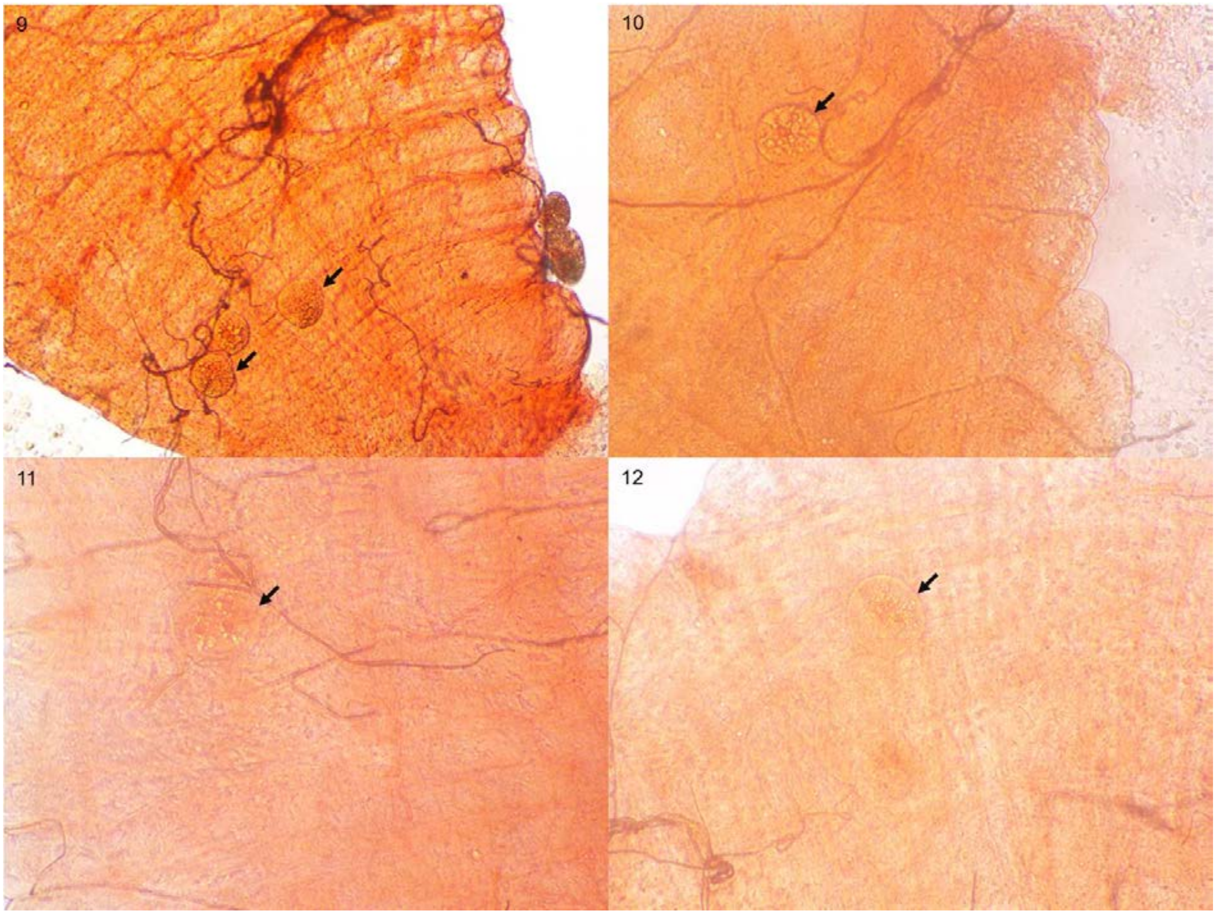
9
10
11
12

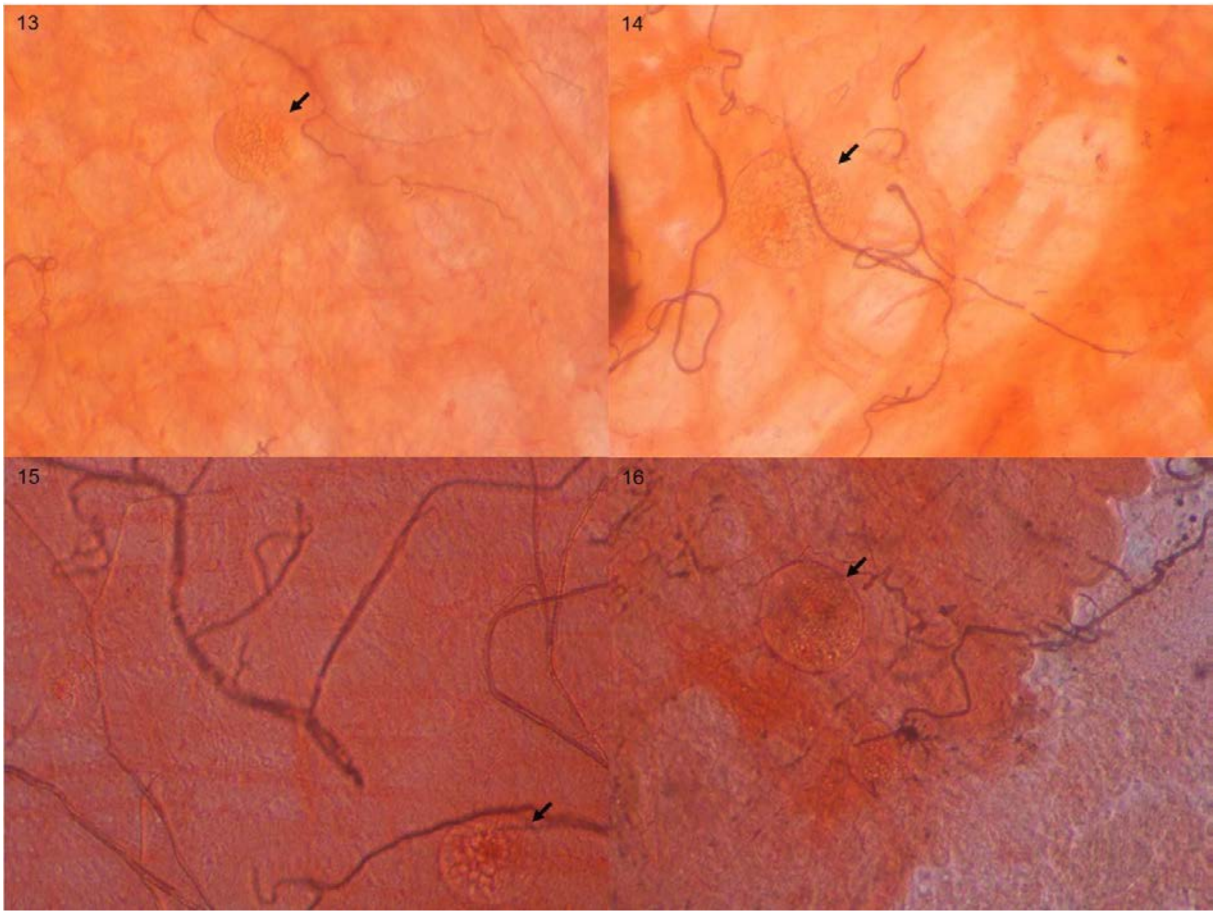
13
14
15
16

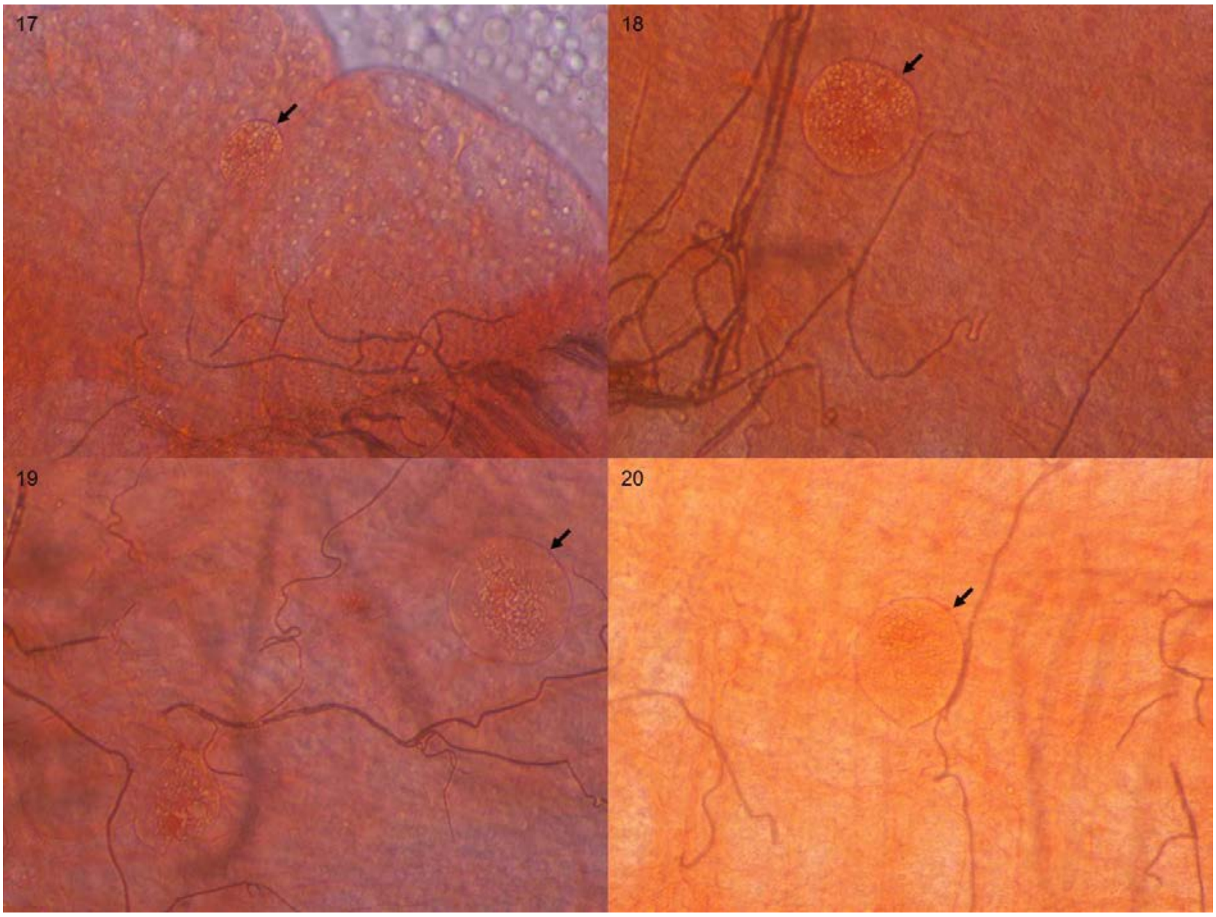
17
18
19
20

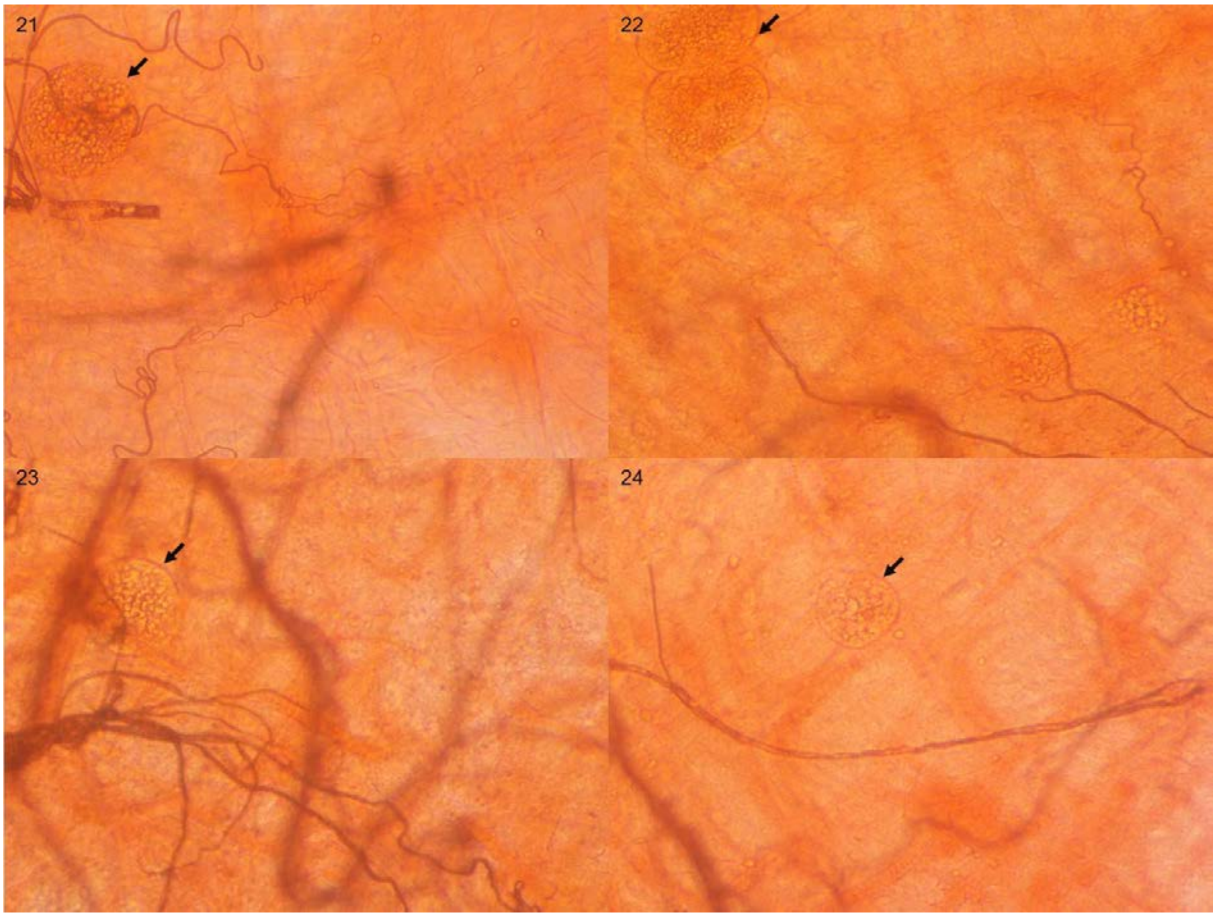
21
22
23
24

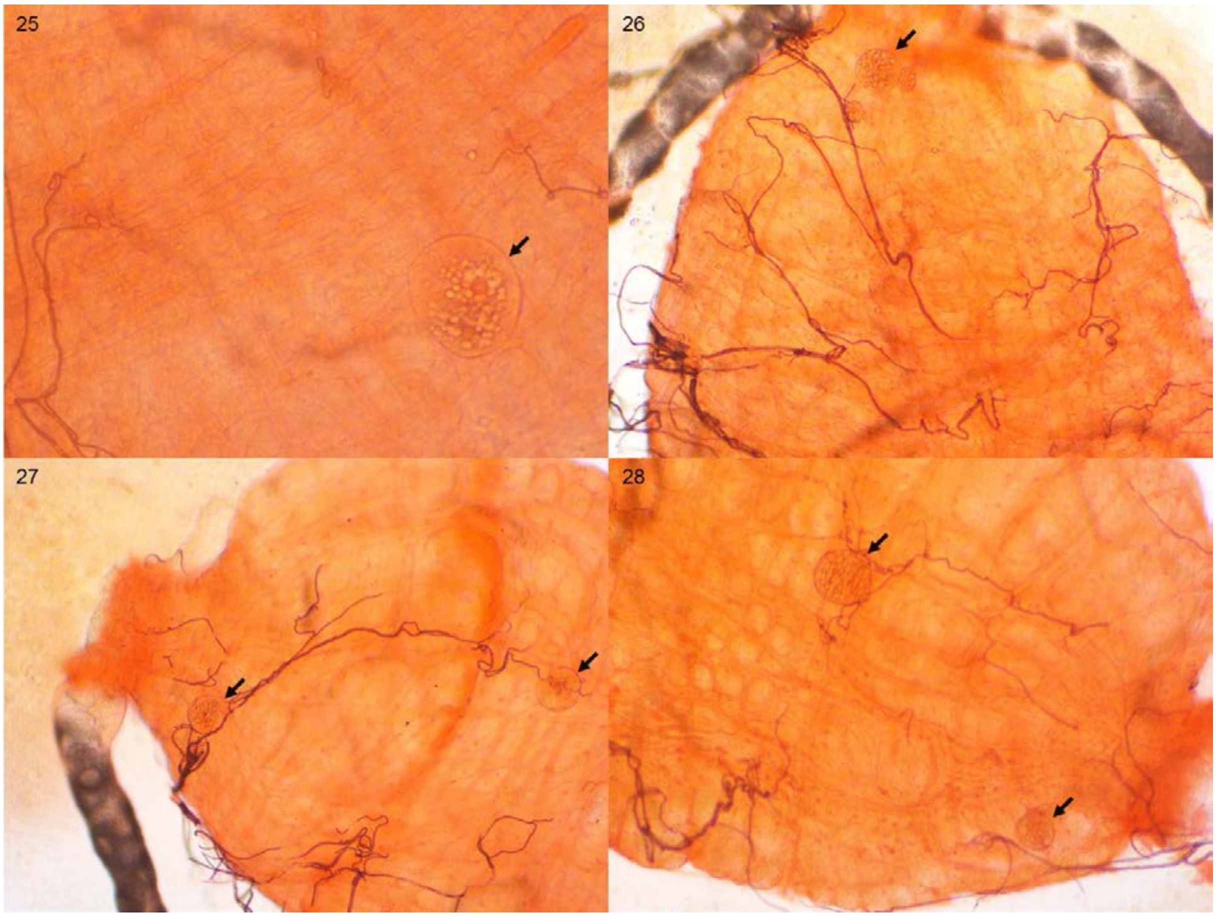
25
26
27
28

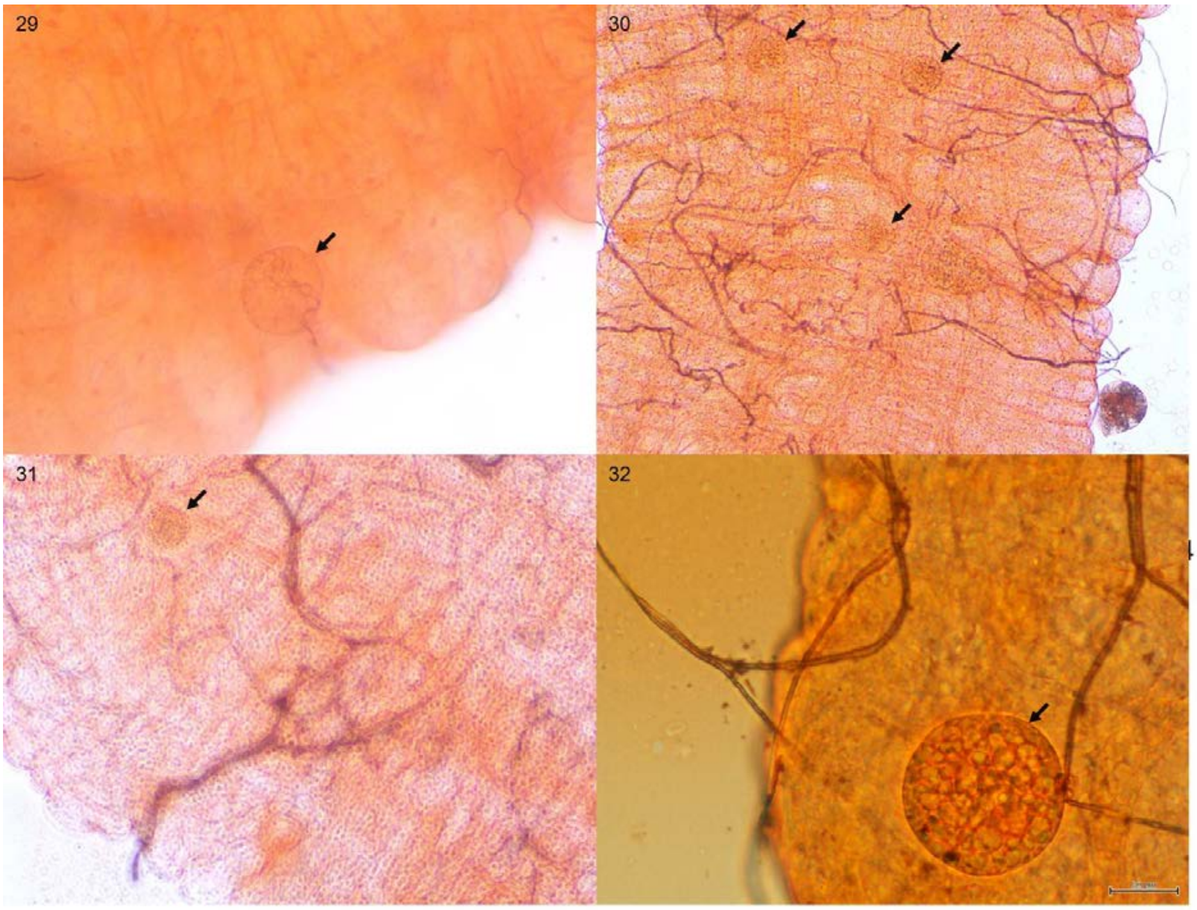
29
30
31
32

(**B**) Representative micrograph images of other ovoid structures observed on mosquito midguts fed with *P. vivax* infected blood (panels 1-16) and non-infected blood (panels 17-24, control mosquitoes)

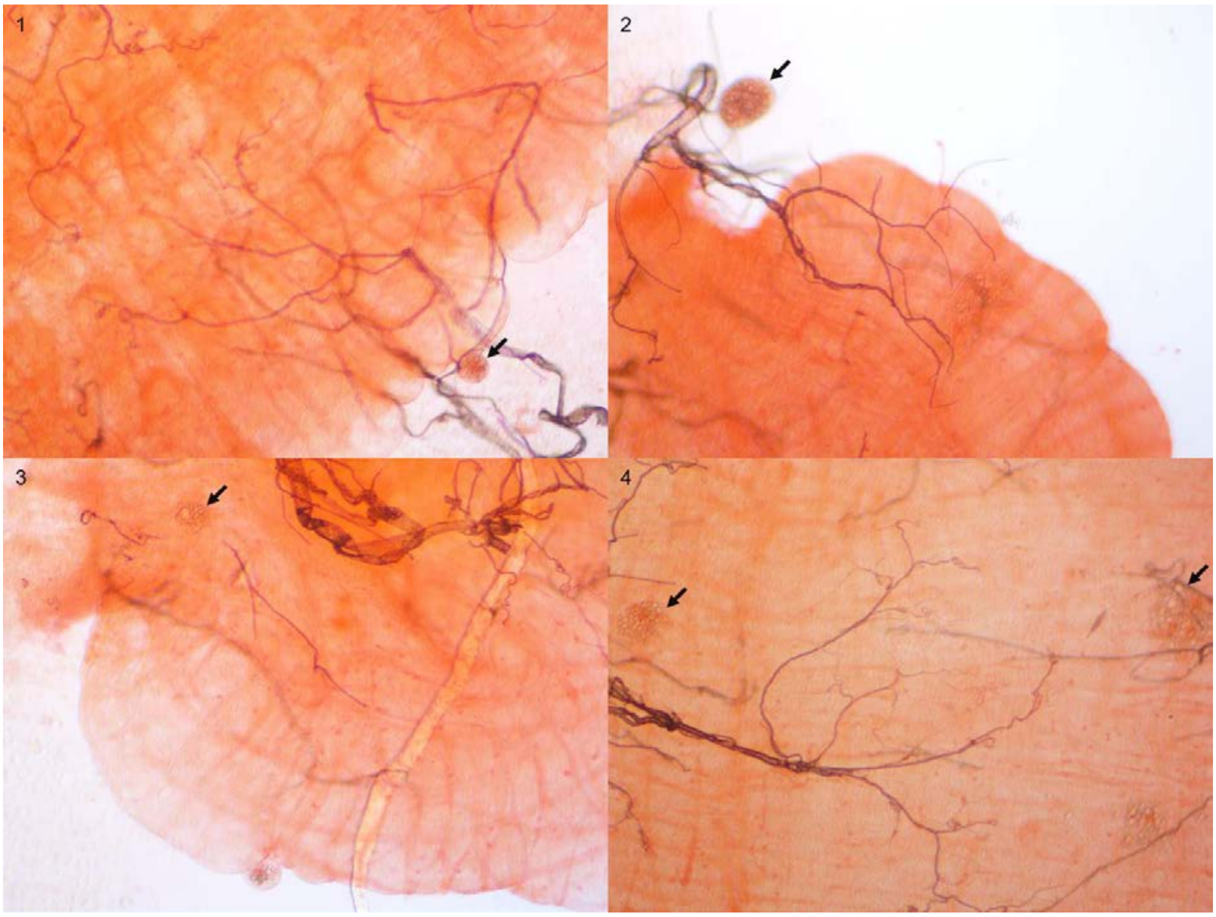

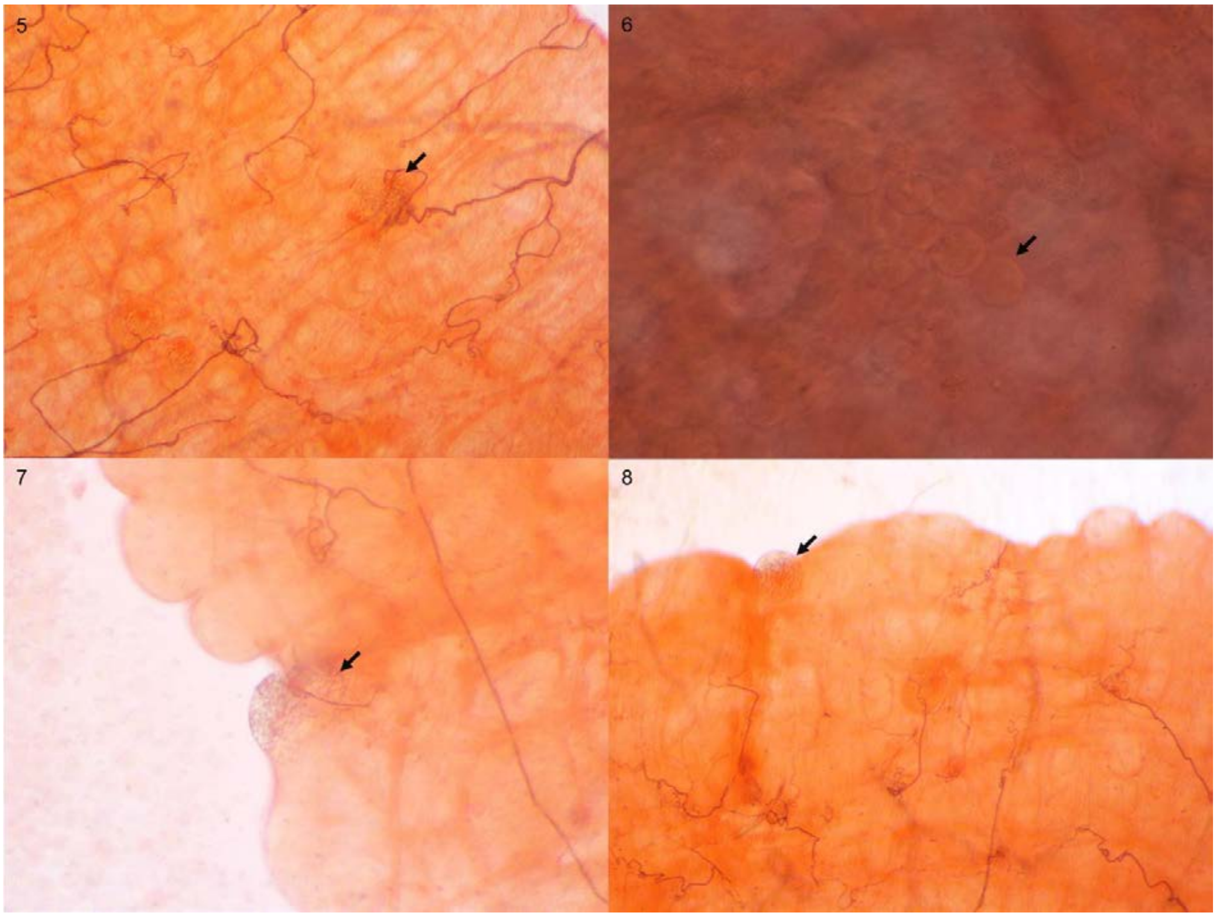
5
6
7
8

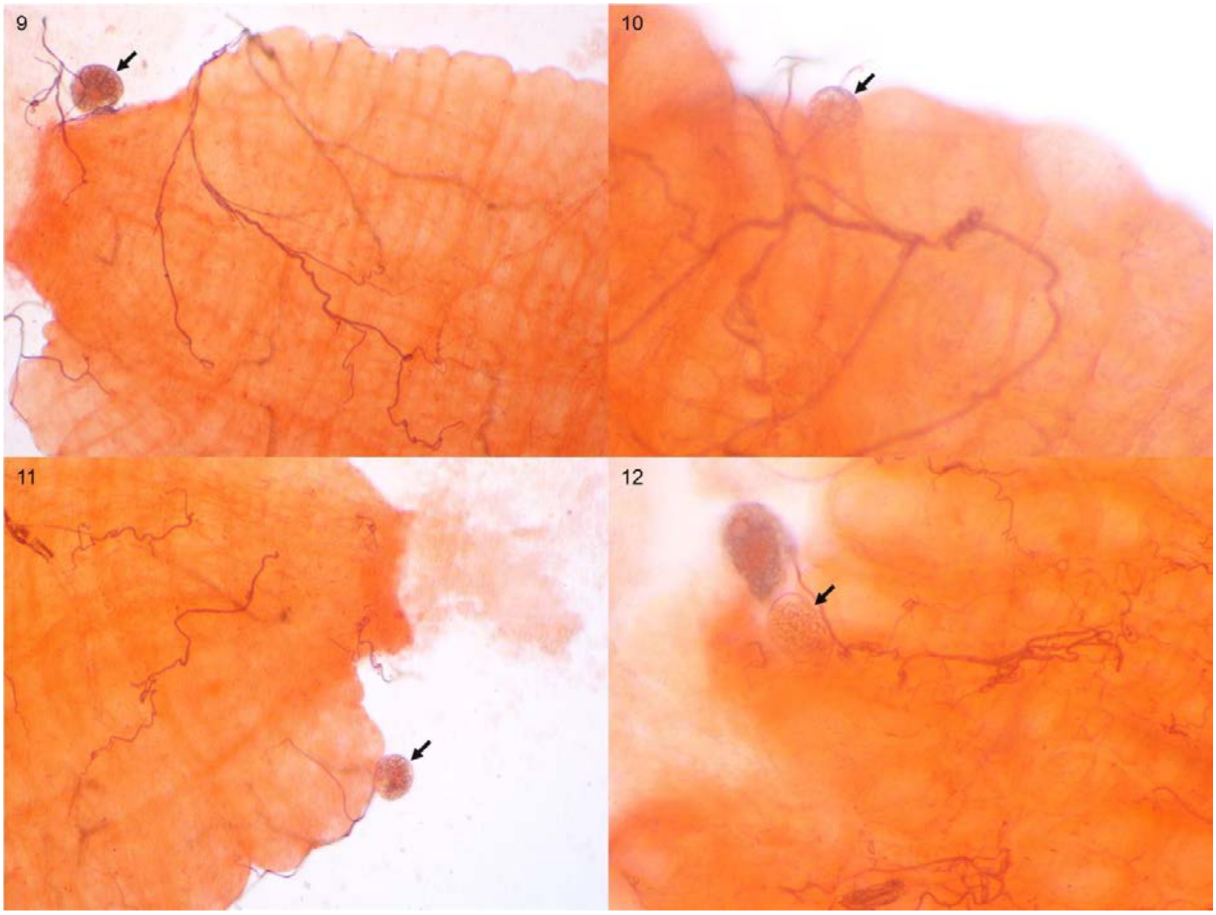
9
10
11
12

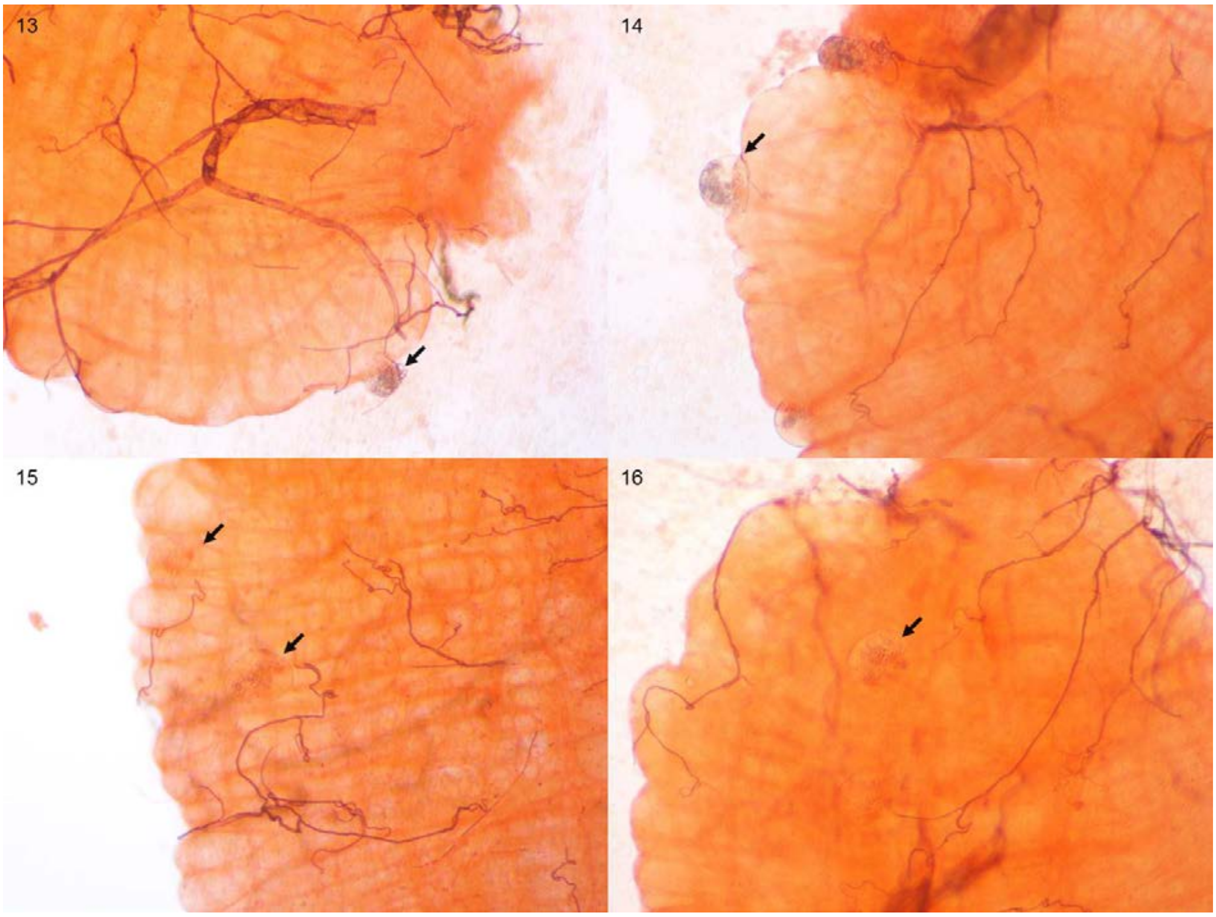
13
14
15
16

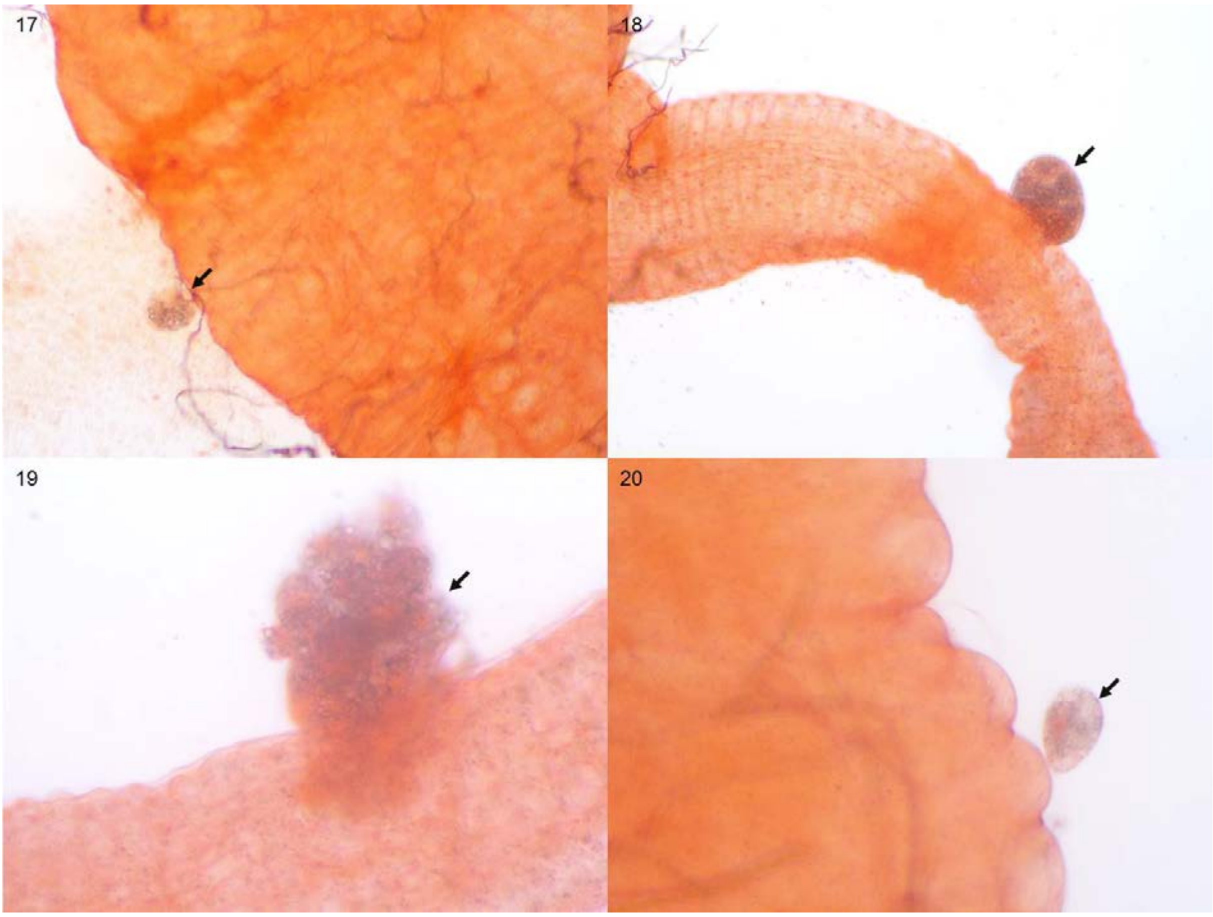
17
18
19
20

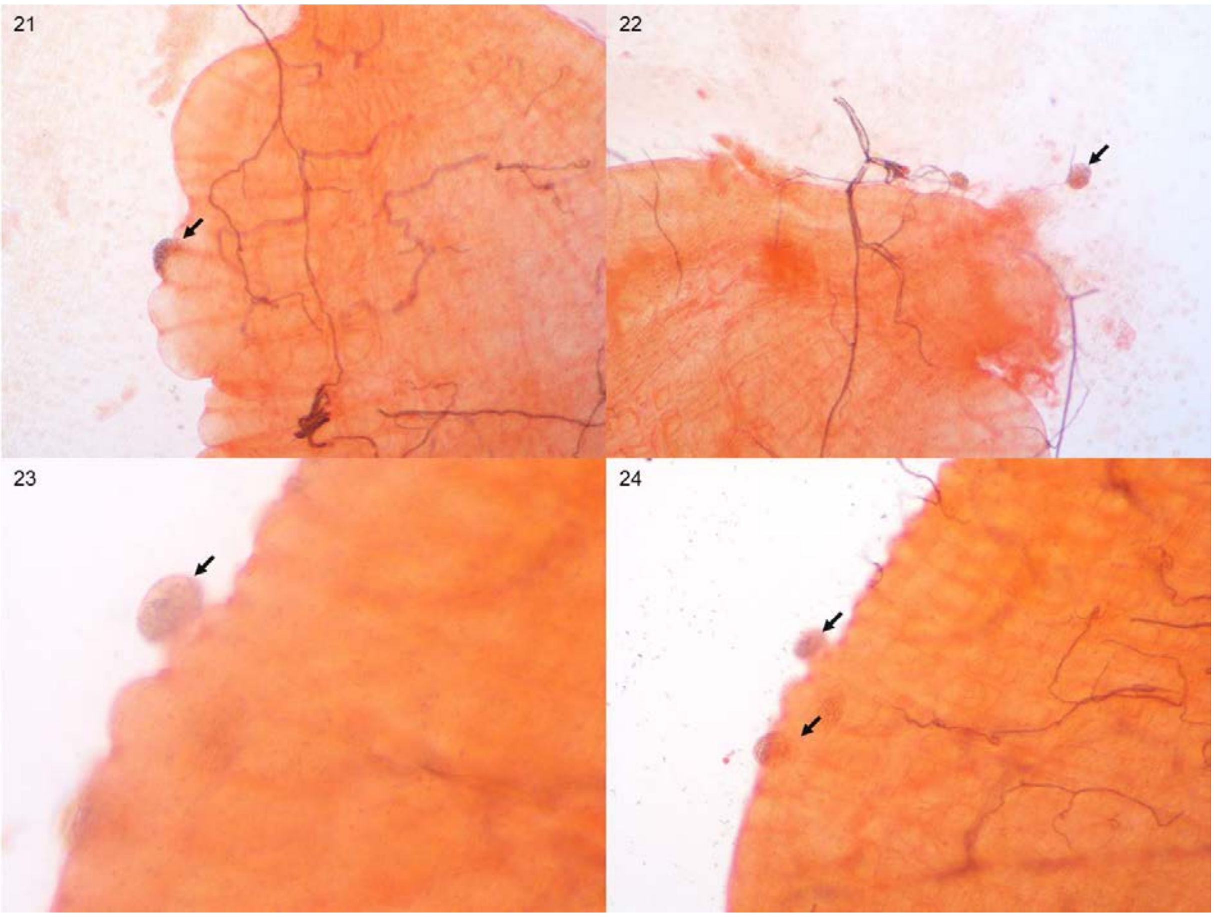
21
22
23
24
