# Supplementary material for: Safety and Reproducibility of a Clinical Trial System Using Induced Blood Stage Plasmodium vivax Infection and Its Potential as a Model to Evaluate Malaria Transmission
Source: PLoS Negl Trop Dis. 2016 Dec 8;10(12):e0005139. doi: 10.1371/journal.pntd.0005139 (PMC5145139; doi:10.1371/journal.pntd.0005139)

S3 Supporting Material. Periodic Acid Schiff (PAS) and Gram chromotrope stained sections of non-*Plasmodium* infected *Anopheles stephensi*. **(A)** PAS stained section through whole *A. stephensi*. Scale bar = 500 µM. **(B)** PAS stained section through midgut. Scale bar = 50 µM. **(C)** and **(D)** Gram Chromotrope stained sections through *A. stephensi* midguts. Scare bar = 50 µM. Arrows show ovoid structures with consistent staining to midgut tissue. h, head. t,thorax. a, abdomen. m, midgut.

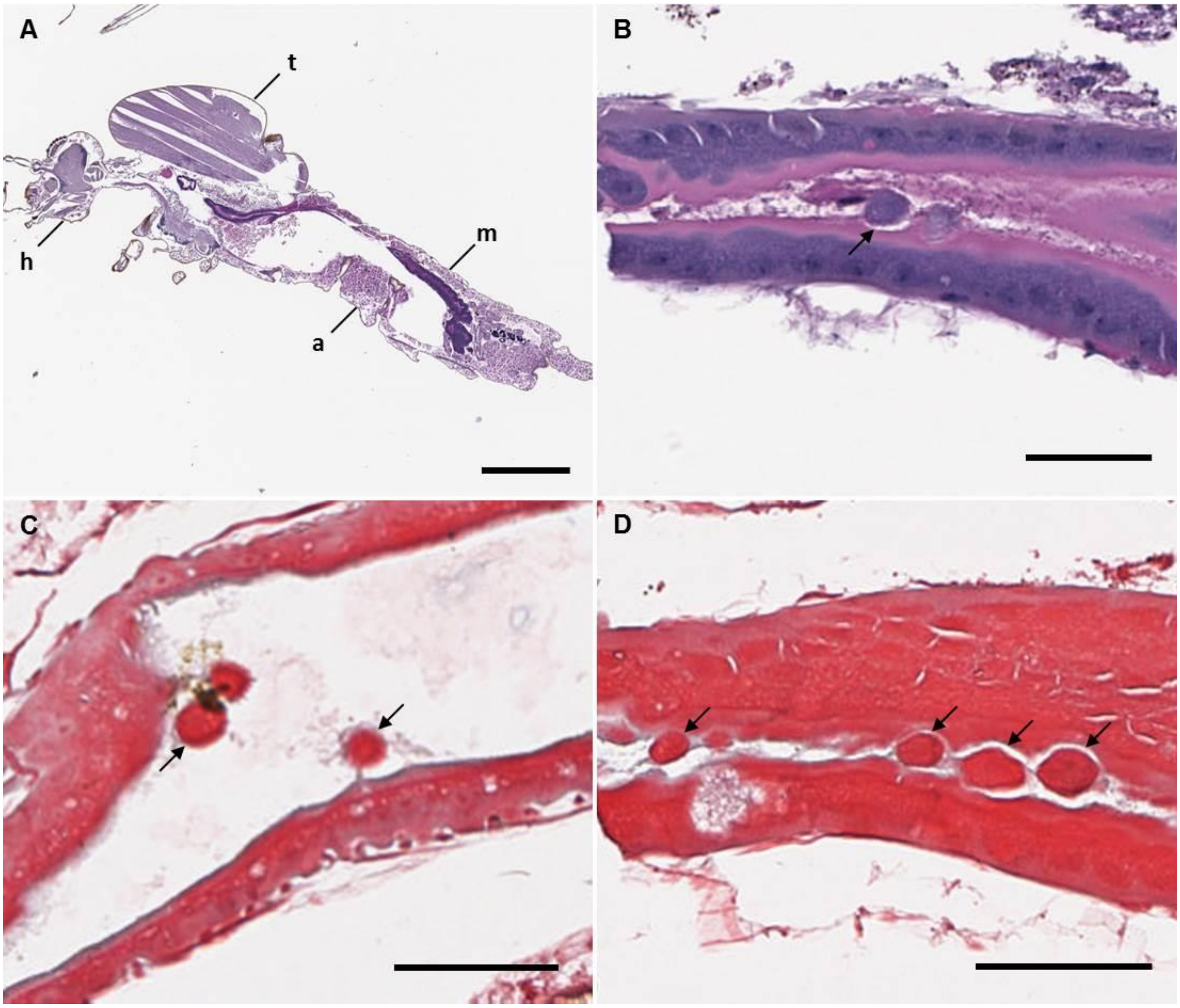

Supplement: S3 Supporting Material — (PDF) [file pntd.0005139.s008.pdf]
